# Puerarin‑Loaded pH‑Responsive ZIF‑90 Nanoparticles Ameliorate Osteoporosis via Enhancing H3K18 Lactylation and Suppressing Aberrant Osteoclast Activation

**DOI:** 10.1002/advs.77869

**Published:** 2026-09-27

**Authors:** Jiajia Lu, Yue Xi, Sheng Wang, Ye Chen, Jiajie Yang, Xiaojian Shi

**Affiliations:** ^1^ Department of Orthopedic Trauma School of Medicine Shanghai Fourth People's Hospital Tongji University Shanghai People's Republic of China; ^2^ Department of Orthopedic Trauma Haimen People's Hospital of Jiangsu Province Haimen People's Republic of China; ^3^ Department of Orthopedic Guangming Traditional Chinese Medicine Hospital of Pudong New Area Shanghai People's Republic of China

**Keywords:** H3K18, histone lactylation, osteoclasts, osteoporosis, puerarin, ZIF‐90 nanoparticles

## Abstract

Osteoporosis is characterized by dysregulated osteoclast activity, leading to excessive bone resorption and structural damage. In this study, we developed pH‐responsive ZIF‐90 nanoparticles loaded with puerarin (PU) (ZIF‐90@PU) for microenvironment‐responsive osteoporosis therapy through epigenetic modulation. Following self‐assembly, the nanoparticles underwent dynamic light scattering (DLS), field emission scanning electron microscopy (FE‐SEM), powder X‐ray diffraction (PXRD), Fourier‐transform infrared spectroscopy (FTIR), and X‐ray photoelectron spectroscopy (XPS); these analyses verified efficient puerarin encapsulation and pH‐triggered release. In cell‐based experiments, ZIF‐90@PU suppressed the differentiation of bone marrow‐derived macrophages (BMMs) into osteoclasts while promoting osteogenic differentiation of bone marrow‐derived mesenchymal stem cells (BMSCs). Administration of ZIF‐90@PU to ovariectomized (OVX) mice produced significant gains in bone mineral density, trabecular architecture, and bone strength without detectable toxicity. Mechanistically, ZIF‐90@PU treatment was associated with increased H3K18 lactylation and reduced NFATc1 signaling, accompanied by suppression of osteoclast activation. This study introduces ZIF‐90@PU as a novel epigenetic‐modulating nanomedicine for osteoporosis treatment.

## Graphical abstract

Schematic illustration of the mechanism by which ZIF‐90@PU alleviates osteoporosis via H3K18la‐mediated NFATc1 pathway suppression.

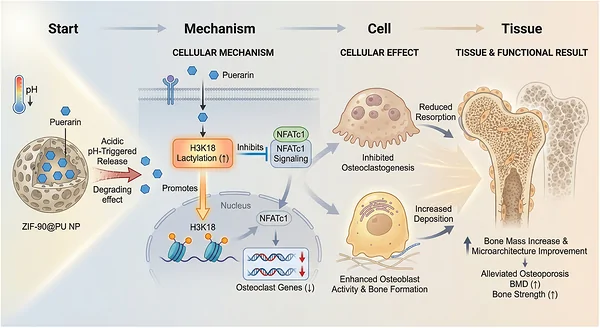

**Abbreviations**
2‐DG: 2‐Deoxy‐D‐Glucose
3D‐µCT: three‐dimensional micro‐computed tomography
ALP: alkaline phosphatase
ALT: Alanine Aminotransferase
ANOVA: analysis of variance
ARS: Alizarin Red S
AST: aspartate aminotransferase
BFR: bone formation rate
BMD: bone mineral density
BMMs: bone marrow‐derived macrophages
BMSCs: bone marrow‐derived mesenchymal stem cells
BP: biological process
BS/BV: bone surface/bone volume
BS/TV: bone surface/total volume
BV/TV: bone volume/total volume
CC: cellular component
CK: creatine kinase
Conn.Dn.: connectivity density
CREA: creatinine
CTX: C‐terminal telopeptide of type i collagen
DEGs: differentially expressed genes
DL%: drug loading
DLS: dynamic light scattering
ECAR: extracellular acidification rate
EE%: encapsulation efficiency
F‐actin: filamentous actin
FBS: fetal bovine serum
FE‐SEM: field emission scanning electron microscopy
GO: gene ontology
H&E: hematoxylin and eosin
H3K18la: H3K18 lactylation
HPLC: high‐performance liquid chromatography
IF: immunofluorescence
IHC: immunohistochemistry
KEGG: Kyoto encyclopedia of genes and genomes
LDH: lactate dehydrogenase
LDHA: lactate dehydrogenase A
MAR: mineral apposition rate
M‐CSF: macrophage colony‐stimulating factor
MF: molecular function
MOF: metal‐organic framework
MOI: multiplicity of infection
OVX: ovariectomized
PBS: phosphate‐buffered saline
PCA: principal component analysis
PCs: principal components
PI: propidium iodide
PU: puerarin
PXRD: powder X‐ray diffraction
RBC: red blood cell
RNA‐seq: RNA sequencing
scRNA‐seq: single‐cell RNA sequencing
SD: standard deviation
Tb.N: trabecular number
TCM: traditional Chinese medicine
TEM: transmission electron microscopy
TRAP: tartrate‐resistant acid phosphatase
UV‐Vis: ultraviolet‐visible
WB: western blot
ZIF‐90@PU: pH‐responsive ZIF‐90 nanoparticles loaded with puerarin

## 1 Introduction

Osteoporosis arises from a persistent imbalance in skeletal remodeling and is characterized by loss of bone mass, deterioration of trabecular architecture, and increased susceptibility to fracture. Its burden continues to expand with population aging, with postmenopausal women being particularly affected [1, 2]. Pharmacological management commonly relies on bisphosphonates, estrogen replacement, and calcitonin, but prolonged treatment may be constrained by adverse effects and poor adherence [3, 4]. These limitations have encouraged the development of strategies that act on disease‐relevant molecular pathways while improving local drug availability. Nanomedicine platforms, including inorganic nanoparticles, polymeric systems, liposomes, and biomimetic vesicles, have therefore been explored for modulation of Wnt/β‐catenin, BMP/Smad, and RANKL/RANK/OPG signaling. A remaining challenge is to achieve responsive drug release within the mildly acidic microenvironment associated with osteoporotic bone [5].

Beyond its classical role as a glycolytic end product, lactate is increasingly recognized as a signaling metabolite that can influence gene regulation through lysine lactylation. This post‐translational modification links cellular metabolic status to epigenetic control and has been implicated in inflammatory disorders, malignancy, and metabolic disease [6, 7, 8]. Histone H3 lysine 18 lactylation (H3K18la) is of particular interest in bone biology because changes in chromatin accessibility and transcription‐factor recruitment may alter osteoclast‐related gene programs [9, 10]. Evidence also indicates that lactylation participates in osteoblast differentiation, osteoclast function, and immune‐mediated regulation of bone homeostasis, potentially in concert with other post‐translational modifications. However, its functional contribution to osteoporosis and its suitability as a pharmacologically tractable epigenetic mechanism remain incompletely defined [10, 11].

Natural bioactive compounds used in traditional Chinese medicine (TCM) provide a broad pharmacological repertoire because they frequently act through multiple molecular targets and signaling pathways [12, 13]. Puerarin (PU), the principal isoflavone of Pueraria lobata, has shown anti‐osteoporotic activity by restraining osteoclastogenesis and supporting osteogenic differentiation [14, 15, 16]. The metabolic and epigenetic events underlying these effects, especially the possible involvement of lactate metabolism and histone lactylation, are still poorly understood. Therapeutic application of PU is also limited by low bioavailability, widespread systemic distribution, and insufficient localization after administration. These properties create a rationale for delivery systems that improve local exposure without altering the established pharmacological activity of PU. Although pH‐responsive puerarin formulations have been investigated in bone‐related settings, their relationship to epigenetic regulation has received comparatively little attention [17].

Metal‐organic frameworks (MOFs) are well suited to stimulus‐responsive drug delivery because their pore structure and loading capacity can be tuned and their frameworks can respond to local physicochemical conditions [18, 19]. Among them, ZIF‐90 combines favorable biocompatibility with acid‐sensitive structural behavior, allowing release of encapsulated cargo as environmental pH decreases [20, 21]. Such properties are relevant to osteoporotic tissue, where a mildly acidic microenvironment may provide a trigger for local drug release. Encapsulation of PU within ZIF‐90 could therefore improve its delivery profile while limiting the shortcomings of conventional systemic exposure. The feasibility of using acid‐responsive ZIF‐family carriers in bone disease is further supported by a ZIF‐8‐based curcumin nanoplatform evaluated in OVX mice [22].

In the present study, we developed a pH‐responsive ZIF‐90@PU system and examined its therapeutic activity and mechanistic effects in cellular and animal models of osteoporosis. Bone structural and histological assessments were combined with single‐cell RNA sequencing (scRNA‐seq), RNA sequencing (RNA‐seq), and LDHA‐intervention experiments to characterize changes in lactate‐associated H3K18 lactylation, NFATc1‐related osteoclast signaling, and bone remodeling. This integrated design was used to determine whether microenvironment‐responsive PU delivery can couple metabolic‐epigenetic regulation with anti‐osteoporotic efficacy.

## 2 Results

### 2.1 Successful Construction of ZIF‐90@PU and Efficient Drug Release Under Acidic Conditions

**FIGURE 1:**
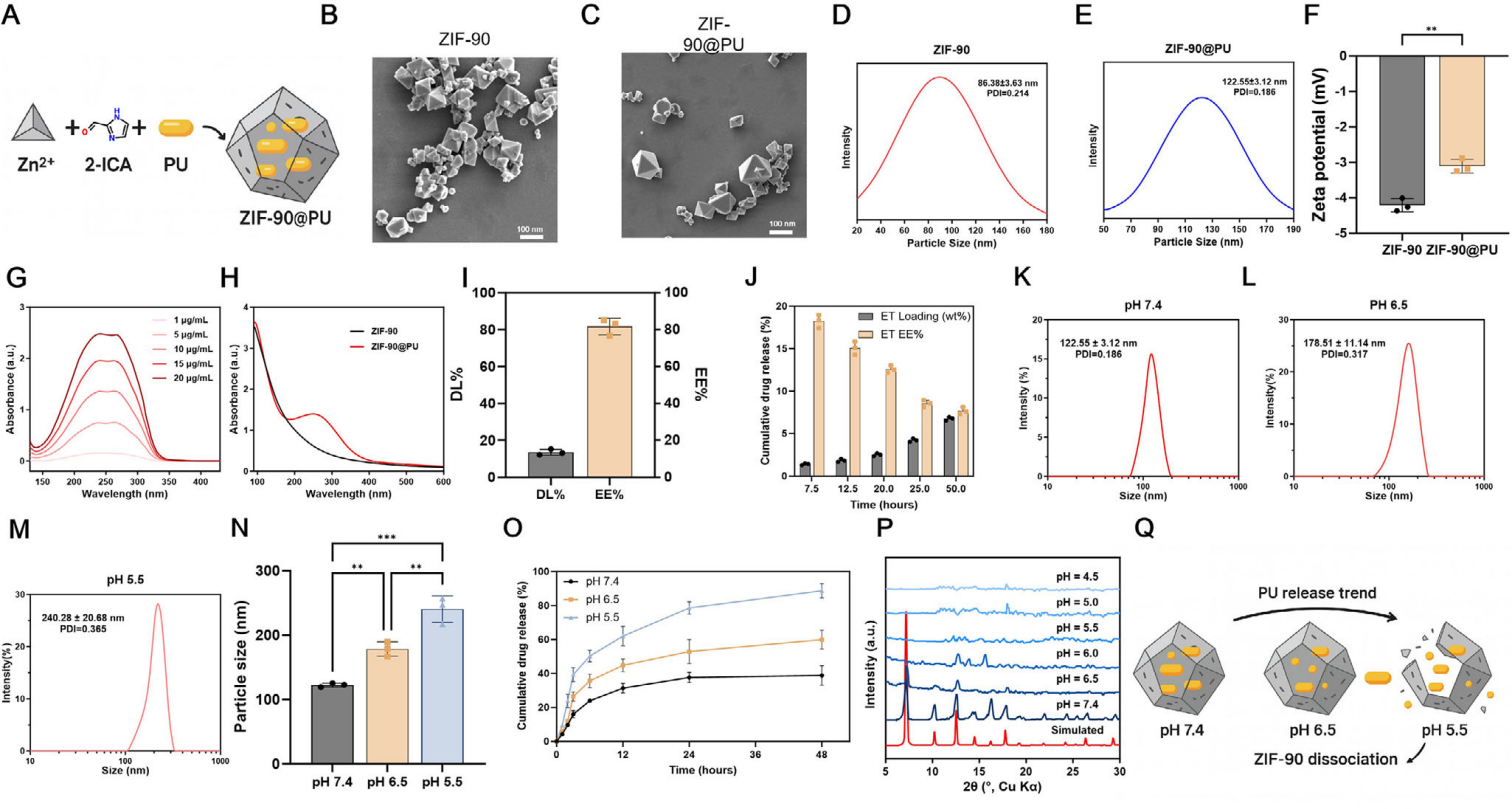
Synthesis and Characterization of ZIF‐90@PU. Note: (A) Schematic illustration of the synthesis process of ZIF‐90@PU, including 2‐ICA (imidazole‐2‐carboxaldehyde); (B,C) FE‐SEM images showing the morphology of ZIF‐90 and ZIF‐90@PU in the dried state; scale bar = 100 nm; (D) DLS analysis of the average particle size distribution of ZIF‐90; (E) DLS analysis of the average particle size distribution of ZIF‐90@PU; (F) Zeta potential analysis of ZIF‐90 and ZIF‐90@PU; (G) UV‐Vis spectra showing the characteristic absorption peak of PU; (H) UV‐Vis spectra of ZIF‐90 and ZIF‐90@PU; (I) DL% and EE% of ZIF‐90@PU; (J) tunability of the ZIF‐90@PU loading content; (K–M) DLS analysis of the particle‐size distributions of ZIF‐90@PU at pH 7.4, 6.5, and 5.5; (N) quantitative comparison of particle sizes under the three pH conditions; (O) PU release curves from ZIF‐90@PU under pH 7.4, 6.5, and 5.5 conditions; (P) PXRD analysis of ZIF‐90 framework stability under different pH conditions; (Q) schematic illustration of the pH‐responsive release mechanism of ZIF‐90@PU. Experiments were repeated three times. **p < 0.01, ***p < 0.001. Data were analyzed using an independent‐samples *t*‐test for two‐group comparisons.

ZIF‐90 was selected as a pH‐responsive carrier to enhance local PU delivery under osteoporotic conditions. The one‐pot preparation route for ZIF‐90@PU is shown in Figure 1A. FE‐SEM images showed that unloaded ZIF‐90 possessed a regular polyhedral structure, while the PU‐loaded particles preserved this overall morphology but displayed a smoother surface (Figure 1B,C). Nanoscale polyhedral particles were also visualized by TEM (Figure S1A). HAADF‐STEM coupled with EDS mapping demonstrated that Zn, C, N, and O were evenly distributed throughout individual particles (Figure S1B). DLS measurements gave a mean diameter of 86.38 ± 2.23 nm for ZIF‐90 and 122.55 ± 3.12 nm after PU loading (Figure 1D,E). Loading also shifted the zeta potential from −4.21 to −3.11 mV (Figure 1F). Nitrogen adsorption‐desorption measurements showed reductions in BET surface area and pore volume after drug incorporation, whereas the main BJH pore‐size peak changed little, consistent with occupancy of the internal pores by PU (Figure S1C,D). Free PU exhibited a characteristic UV‐Vis absorption peak at 250 nm (Figure 1G), and the same feature appeared in ZIF‐90@PU but not in blank ZIF‐90 (Figure 1H). Together with the decrease in accessible surface area and pore volume and the preserved framework‐related PXRD profile, these observations are consistent with incorporation of PU into the ZIF‐90 framework. Drug‐loading and encapsulation efficiencies were 13.56% and 81.65%, respectively (Figure 1I). FTIR and XPS were then used to further characterize drug‐framework interactions (Figure S1E,F). ZIF‐90@PU retained the major framework‐associated FTIR bands while gaining PU‐related O─H and C─O/C─O─C features, with additional changes in peak position, width, and intensity in the O─H, C═O/C═N, and C─O/C─O─C regions. XPS likewise preserved the Zn 2p and N 1s signals but showed stronger oxygen‐containing C 1s/O 1s components after loading, further supporting PU incorporation within the framework.

The influence of PU input on loading performance was evaluated using initial feed amounts of 2, 4, 8, and 16 mg. Increasing the feed progressively raised DL%, which reached 13.56% at 8 mg and changed only modestly after the feed was increased to 16 mg, indicating that the available loading capacity was approaching saturation (Figure 1J). EE% remained above 85% at the lower inputs and declined slightly as more PU was added. Thus, adjustment of the initial PU/Zn2+ ratio allowed the loading content to be varied over an approximate range of 4%–15%.

The pH response of ZIF‐90@PU was next examined by DLS and release measurements. Particle size changed little at pH 7.4, increased at pH 6.5, and became larger still at pH 5.5 (Figure 1K–N). PU release followed the same acidity‐dependent pattern, remaining limited under physiological pH, accelerating at pH 6.5, and increasing further at pH 5.5 (Figure 1O). After 24 h in PBS at pH ≤ 6.0, the characteristic PXRD reflection at 2θ = 7° was lost, indicating disruption of the framework (Figure 1P), whereas the crystalline structure was retained above pH 6.0.

These characterization data establish that ZIF‐90@PU was successfully prepared as a well‐dispersed crystalline formulation with adjustable drug loading. Its acid‐sensitive structural response provides a basis for controlled PU release within the mildly acidic osteoporotic microenvironment (Figure 1Q).

### 2.2 ZIF‐90@PU Exhibits Favorable Biosafety and Time‐Dependent Bone Accumulation

**FIGURE 2:**
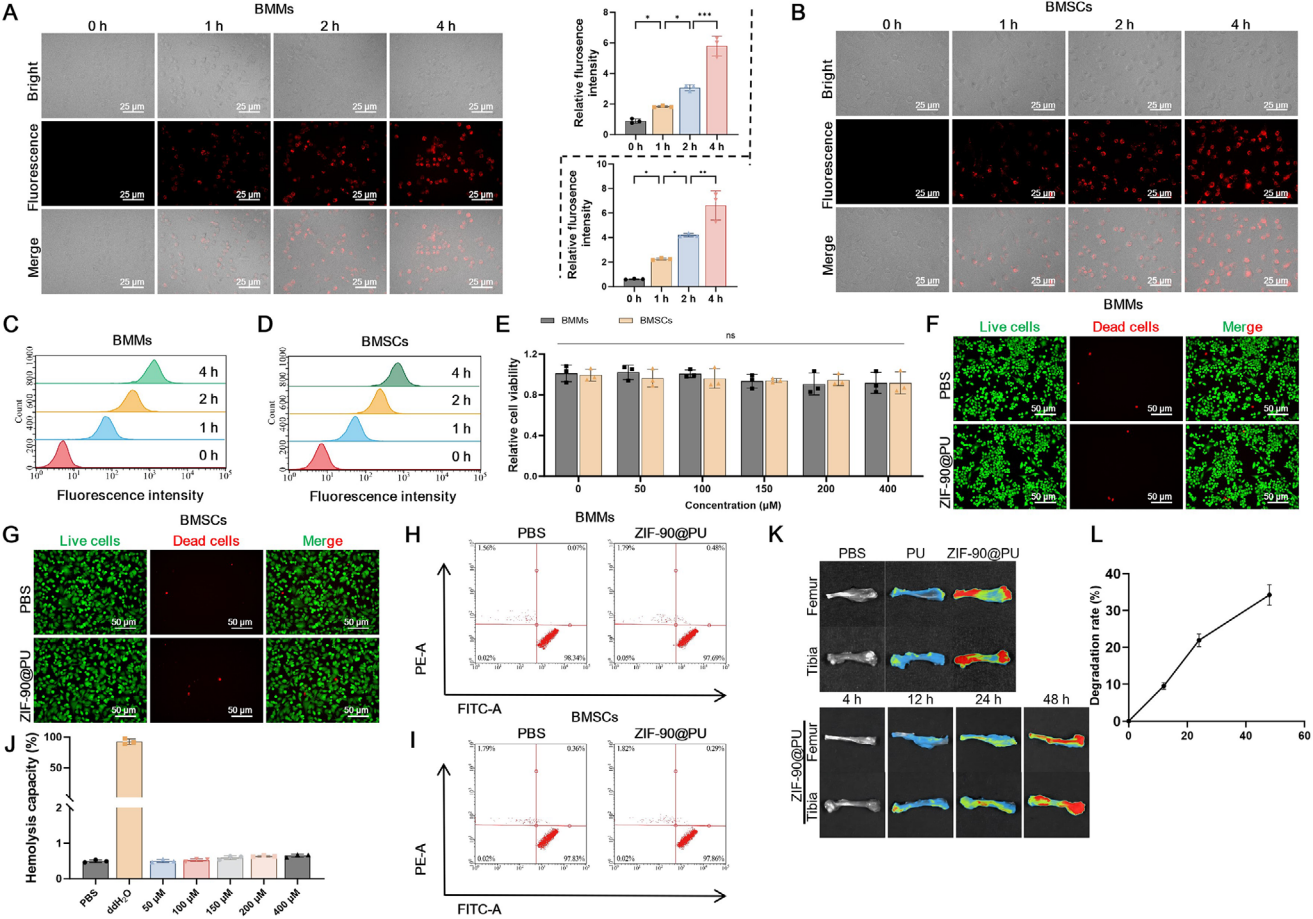
In vitro cellular uptake, biosafety, and time‐dependent bone accumulation of ZIF‐90@PU. Note: (A,B) IF staining showing the uptake of Cy5.5‐labeled ZIF‐90@PU by BMSCs and BMMs; scale bar = 25 µm; (C,D) flow cytometry quantification of ZIF‐90@PU uptake by BMSCs and BMMs; (E) CCK‐8 assay evaluating cell viability after treatment with different concentrations of ZIF‐90@PU; (F,G) live/dead staining after ZIF‐90@PU treatment; scale bar = 50 µm; (H,I) flow cytometric analysis of cell survival after ZIF‐90@PU treatment; Q2‐UL, Calcein AM^−^/PI^+^ dead cells; Q2‐LL, Calcein AM^−^/PI^−^ debris; Q2‐LR, Calcein AM^+^/PI^−^ live cells; (J) hemolysis assay using C57BL/6 mouse red blood cells treated with different concentrations of ZIF‐90@PU; (K) biodistribution of DiR‐labeled ZIF‐90@PU in leg bones of OVX mice; (L) time‐dependent degradation rate of DiR‐labeled ZIF‐90@PU (*n* = 3 per time point). Cell experiments were repeated three times. **p < 0.01, ***p < 0.001, ****p < 0.0001, ns, not significant. Data were analyzed using one‐way ANOVA with Tukey's HSD post hoc test for multiple‐group comparisons and independent‐samples *t*‐test for two‐group comparisons.

Cellular uptake and cytocompatibility were first examined in BMSCs and BMMs. After exposure to Cy5.5‐labeled ZIF‐90@PU, intracellular fluorescence increased progressively from 0 to 1, 2, and 4 h in both cell types, demonstrating time‐dependent uptake (Figure 2A,B). Flow‐cytometric measurements showed the same pattern and confirmed a significant increase in fluorescence at 4 h compared with baseline (Figure 2C,D).

Cell viability remained stable over the tested ZIF‐90@PU concentration range in the CCK‐8 assay for both BMSCs and BMMs (Figure 2E). Consistently, live/dead staining showed no appreciable increase in PI‐positive cells after nanoparticle exposure (Figure 2F,G), and flow cytometry produced comparable viability results (Figure 2H,I). Blood compatibility was evaluated separately using mouse erythrocytes. Across all tested concentrations, ZIF‐90@PU produced no visible hemolysis relative to the negative control (Figure 2J), supporting favorable in vitro biocompatibility.

Biodistribution was then assessed in OVX mice. Bilateral ovariectomy produced pronounced uterine atrophy, confirming model establishment (Figure S2A,B). Following intravenous administration of DiR‐labeled ZIF‐90@PU, tissues were harvested at 4, 12, 24, and 48 h. Fluorescence was initially concentrated in the liver and spleen, whereas bone signals were weak; with time, signals in these organs declined while fluorescence in bone became more prominent, including clear accumulation in trabecular regions by 48 h (Figure 2K). Quantitative analysis confirmed this decline in liver and spleen fluorescence. In the liver, the 24‐h signal was lower than that at 4 h (*p <* 0.01), the 48‐h signal was lower than that at 4 h (*p <* 0.001), and the 48‐h value was also lower than that at 12 h (*p <* 0.01). In the spleen, corresponding reductions were observed for 4 h versus 24 h (*p <* 0.05), 4 h versus 48 h (*p <* 0.01), and 12 h versus 48 h (*p <* 0.05) (Figure S2D). The calculated degradation index increased over time (Figure 2L). At 48 h, fluorescence in bone exceeded that in the heart, lung, and kidney and was similar to the liver signal; ZIF‐90@PU also accumulated in bone more strongly than free PU. The distribution pattern therefore supports time‐dependent bone accumulation rather than active ligand‐mediated targeting.

Taken together, the formulation showed efficient uptake by both tested cell types, low in vitro toxicity, and progressive accumulation in bone after systemic administration. Bone‐marrow distribution analyses further indicated uptake by several resident cell populations, with the strongest signal in the monocyte/macrophage lineage.

### 2.3 ZIF‐90@PU Significantly Alleviates Osteoporosis and Exhibits Favorable Safety

**FIGURE 3:**
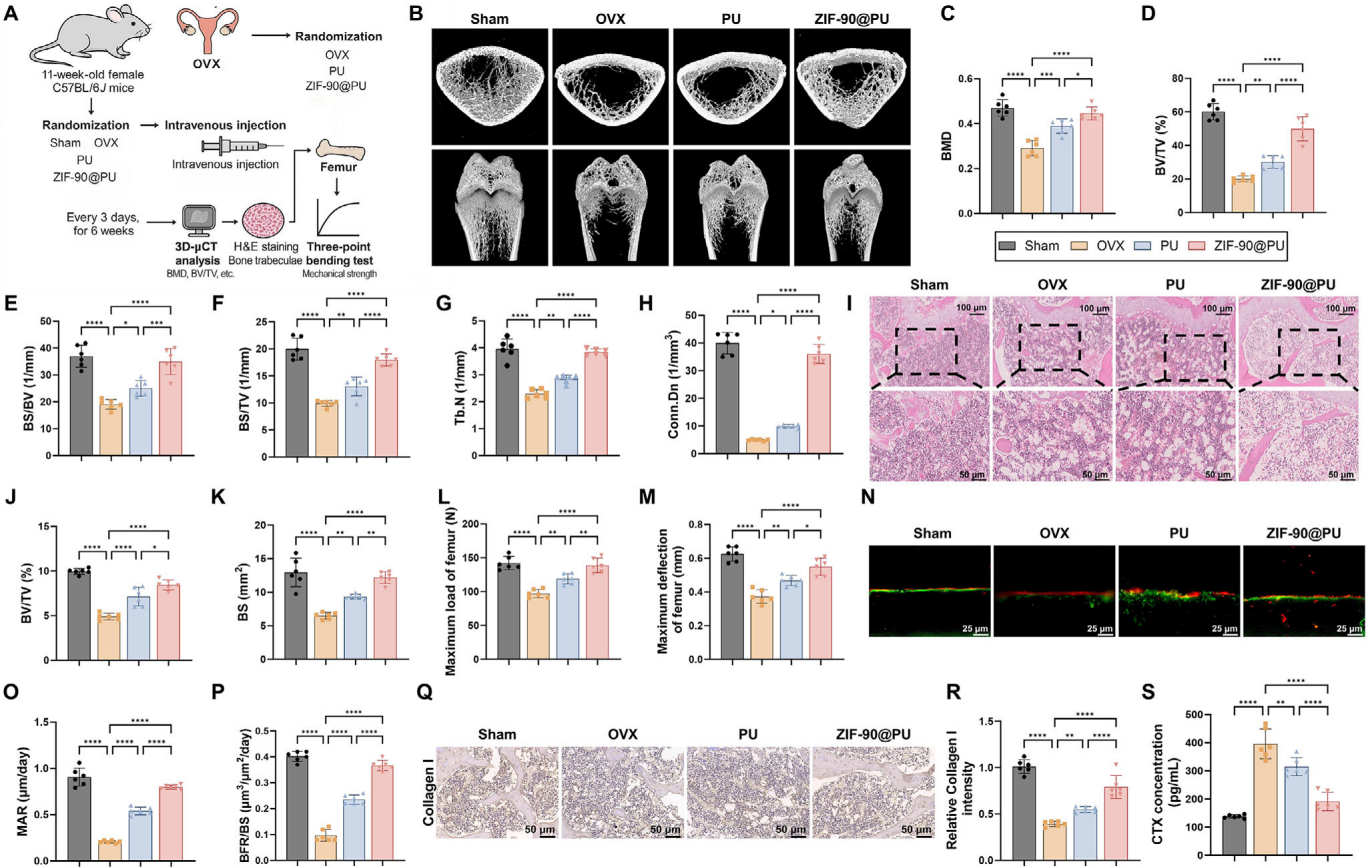
Therapeutic efficacy of ZIF‐90@PU in osteoporotic mice. Note: (A) Schematic representation of the in vivo experimental procedure for evaluating the therapeutic efficacy of ZIF‐90@PU; (B) 3D‐µCT reconstruction of femoral bone microarchitecture in each group; (C–H) quantitative 3D‐µCT analysis of BMD, BV/TV, BS/BV, BS/TV, Tb.N, and Conn.Dn; (I–K) H&E staining of femoral trabecular bone and quantification of bone parameters (BV/TV and BS); scale bars = 100 and 50 µm; (L,M) three‐point bending test of the maximum load and maximum deflection of the femur; (N–P) in vivo calcein/ARS double‐fluorescence labeling for the MAR and BFR; (Q,R) IHC staining of Collagen I expression in mouse femoral tissue (scale bar = 50 µm); (S) serum CTX levels measured by ELISA. Six animals were included in each group. *p < 0.05, **p < 0.01, ***p < 0.001, ****p < 0.0001 between groups. Data were analyzed using one‐way ANOVA with Tukey's HSD post hoc test.

Therapeutic efficacy was evaluated after six weeks of treatment in Sham, OVX, PU, and ZIF‐90@PU groups (Figure 3A). Three‐dimensional µCT showed extensive trabecular loss after OVX, partial preservation after free PU treatment, and greater structural recovery with ZIF‐90@PU (Figure 3B). Consistent with the reconstructions, OVX reduced BMD, BV/TV, BS/BV, BS/TV, Tb.N, and Conn.Dn relative to Sham. Each of these indices improved with PU, and all were further increased in the ZIF‐90@PU group compared with PU alone (Figure 3C–H), indicating a stronger improvement in bone mass and microarchitecture with the nanodrug.

H&E sections provided parallel histological evidence. OVX produced sparse, widely separated trabeculae and lower BS and BV/TV than Sham. Free PU partially restored trabecular organization and increased both quantitative indices, whereas ZIF‐90@PU produced a denser and more orderly trabecular network with additional increases in BV/TV and BS (Figure 3I–K).

Mechanical testing showed that OVX femurs had lower maximum load and deflection than Sham femurs. Both parameters increased after PU administration and rose further after ZIF‐90@PU treatment (Figure 3L,M). Dynamic bone formation was assessed by calcein/ARS double labeling. MAR and BFR were reduced by OVX, partially recovered with PU, and further elevated in the ZIF‐90@PU group (Figure 3N–P), consistent with enhanced new bone formation.

Type I collagen staining also decreased after OVX and increased after treatment, with the strongest collagen I‐positive signal in the ZIF‐90@PU group relative to free PU (Figure 3Q,R). Serum CTX showed the opposite pattern: levels rose in OVX mice, declined after PU treatment, and were reduced further by ZIF‐90@PU (Figure 3S). In conjunction with the µCT findings, these histological and biochemical changes indicate simultaneous enhancement of bone formation and suppression of bone resorption.

Safety was assessed throughout the treatment period (Figure S2E). Body weight trajectories were similar among groups (Figure S2F), and H&E examination of the heart, liver, spleen, lung, and kidney revealed no obvious treatment‐related lesions (Figure S2G). Serum ALT, AST, BUN, CREA, LDH, and CK also remained within comparable ranges (Figure S2H–M). Cellular localization within bone marrow was examined 48 h after injection. DiR‐ZIF‐90@PU was detected in CD11b+ monocyte/macrophage‐lineage cells, Ly6G+ neutrophils, and CD31+ endothelial cells, with the highest mean fluorescence intensity in the CD11b+ population (Figure S2O); representative fluorescence images are shown in Figure S2N. Thus, the therapeutic improvement in bone structure and mechanics occurred without evident systemic toxicity under the tested conditions.

### 2.4 Sequencing Analyses Indicate That ZIF‐90@PU Remodels Bone Marrow Cell Populations and Suppresses Glycolysis and Lactate Metabolism in Osteoclasts

**FIGURE 4:**
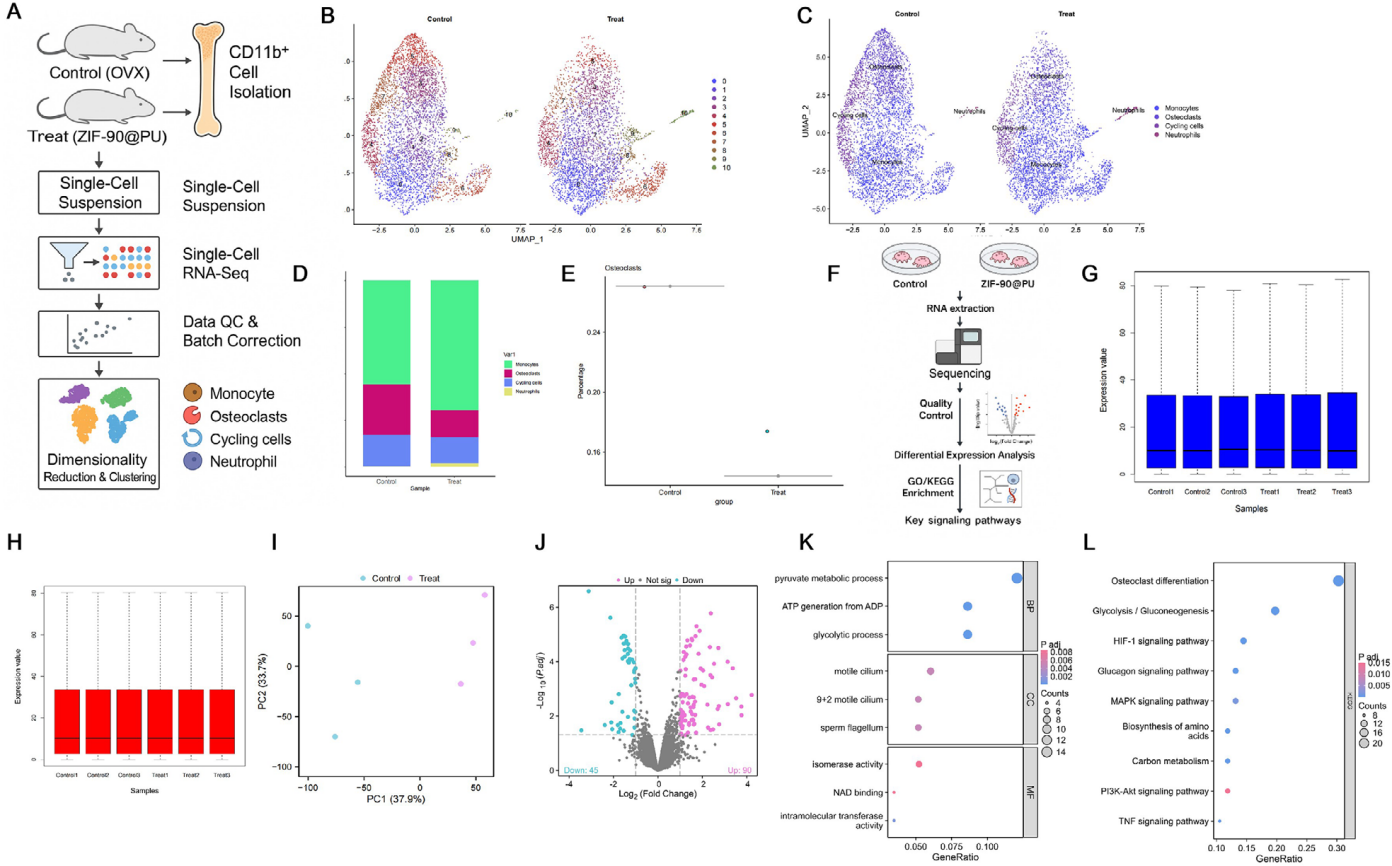
Regulation of bone marrow CD11b^+^ Cell populations and the osteoclast transcriptome by ZIF‐90@PU. Note: (A) Schematic diagram outlining the workflow of scRNA‐seq analysis; (B) UMAP clustering visualization showing cellular aggregation and distribution, with each color representing a distinct cluster; (C) UMAP dimensionality reduction plot identifying four major cell populations within bone marrow CD11b^+^ cells; (D) Quantification of different cell types in the Control and Treat groups; (E) Proportional comparison of osteoclasts between the Control and Treat groups; (F) Schematic diagram of the experimental workflow for RNA‐seq and data analysis; (G,H) Quality control and normalization of high‐throughput transcriptomic sequencing data, showing sample distributions before normalization (G) and after quantile normalization (H); (I) PCA illustrating differences in gene expression patterns of osteoclasts between the Control and ZIF‐90@PU groups; (J) Volcano plot analysis of DEGs, depicting the distribution of upregulated and downregulated genes in osteoclasts; (K) GO enrichment analysis identifying biological processes enriched among DEGs; (L) KEGG pathway enrichment analysis showing the signaling pathways associated with DEGs.

scRNA‐seq was used to describe the cellular composition of bone marrow CD11b+ cells in the osteoporosis model (Figure 4A). Samples were obtained from one OVX control mouse and one ZIF‐90@PU‐treated mouse (*n* = 1 per group); accordingly, the single‐cell data were used for descriptive cell‐state characterization rather than group‐level inference. Quality filtering was based on nFeature_RNA, nCount_RNA, and percent.mt (Figure S3A). After filtering, nCount_RNA correlated negatively with percent.mt (*r* = −0.2) and positively with nFeature_RNA (*r* = 0.9) (Figure S3B). Cell‐cycle states were assigned with CellCycleScoring (Figure S3C), and PC ranking showed that PC_1‐PC_30 retained most of the variance among highly variable genes (Figure S3D). Harmony processing reduced sample‐associated batch structure (Figure S3E–G).

UMAP dimensionality reduction was performed with the top 30 PCs, and clustree was used to inspect cluster stability across resolutions (Figure S4A). Eleven clusters were retained in the final solution (Figure 4B and Figure S4B). Annotation using established marker genes, published information, and CellMarker references grouped these clusters into monocytes, osteoclasts, cycling cells, and neutrophils (Figure 4C).

Marker expression supported these annotations: Lgmn, Cebpb, and Csf1r characterized monocytes; Mmp9, Acp5, and Ctsk marked osteoclasts; Cdk1, Mki67, and Top2a identified cycling cells; and Ngp, Camp, and S100a9 defined neutrophils (Figure S4C). Descriptive comparison of cell composition showed a lower osteoclast‐related fraction in the treated sample (Figure 4D,E, Figure S4D). This pattern was directionally consistent with the independent TRAP and MMP9/NFATc1 measurements.

Bulk transcriptomic profiling was subsequently performed in osteoclasts from PBS‐treated Control and ZIF‐90@PU‐treated groups (Figure 4F). After sequencing, normalization reduced between‐sample distributional differences (Figure 4G,H). PCA separated the two treatment conditions in expression space (Figure 4I), indicating broad transcriptional remodeling after ZIF‐90@PU exposure.

Using |log_2_FC| > 1 and *p <* 0.05, limma identified 135 DEGs, comprising 90 upregulated and 45 downregulated genes (Figure 4J). GO enrichment highlighted terms including pyruvate metabolism, motile cilium, and isomerase activity (Figure 4K). KEGG analysis identified osteoclast differentiation, glycolysis/gluconeogenesis, and HIF‐1 signaling among the enriched pathways (Figure 4L), linking the transcriptional response to metabolic programs relevant to lactate production and osteoclast function.

Overall, the single‐cell dataset suggested a treatment‐associated shift in bone marrow CD11b+ cell composition, whereas RNA‐seq demonstrated substantial changes in osteoclast transcriptional programs. These observations were concordant with the independent histological and molecular evidence for altered glycolysis, lactate metabolism, and osteoclast activity.

### 2.5 ZIF‐90@PU Significantly Suppresses Osteoclast Activation and Bone‐Resorption Pathways

**FIGURE 5:**
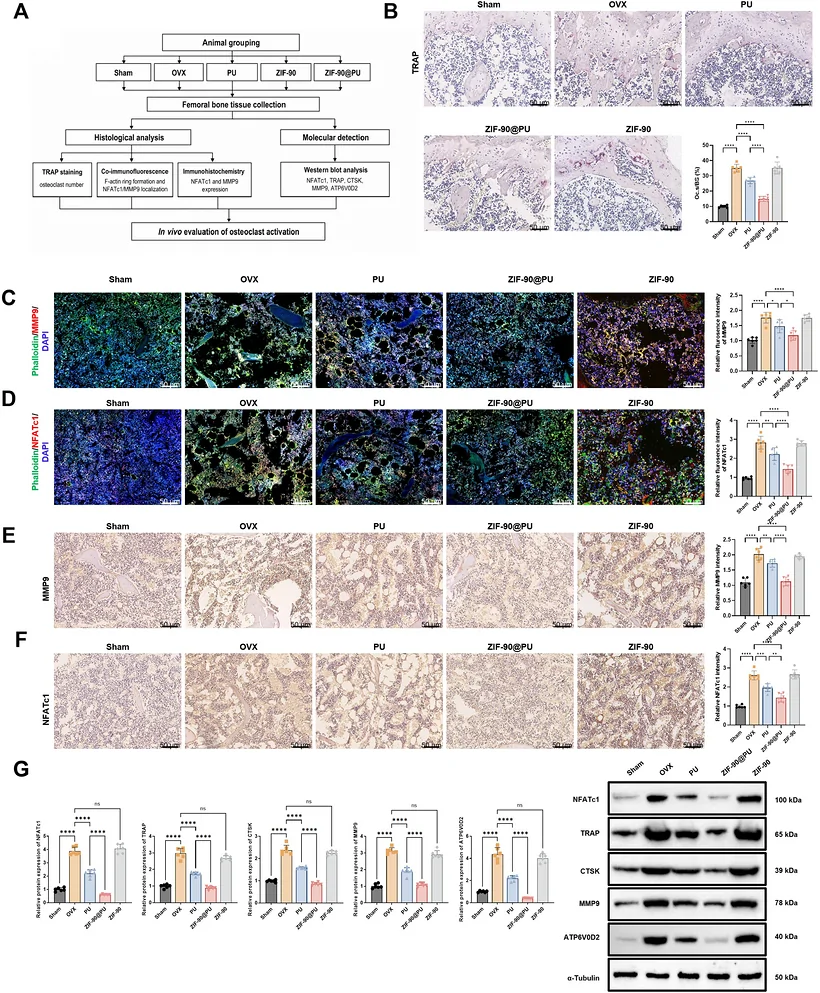
ZIF‐90@PU‐mediated suppression of osteoclast activation and associated protein expression. Note: (A) Schematic diagram outlining the workflow for histological and molecular analyses; (B) TRAP staining of femoral sections to evaluate osteoclast numbers; scale bar = 50 µm; (C,D) co‐IF staining using phalloidin with MMP9 and NFATc1 antibodies to assess F‐actin ring formation and MMP9/NFATc1 signals; scale bar = 50 µm; (E,F) IHC staining of NFATc1 and MMP9 in femoral tissue; scale bar = 50 µm; (G) Western blot analysis of osteoclast‐associated proteins (NFATc1, TRAP, CTSK, MMP9, and ATP6V0D2). n = 6 animals per group. **p < 0.01, ***p < 0.001, ****p < 0.0001. Data were analyzed using one‐way ANOVA with Tukey's HSD post hoc test.

The in vivo osteoclast phenotype was examined across Sham, OVX, PU, blank ZIF‐90, and ZIF‐90@PU groups (Figure 5A). TRAP staining showed a pronounced increase in multinucleated osteoclasts after OVX. Free PU reduced TRAP‐positive cell numbers, and ZIF‐90@PU produced a further reduction, while blank ZIF‐90 remained similar to OVX (Figure 5B).

Because intact F‐actin rings form the sealing zone required for osteoclastic resorption [23], cytoskeletal organization was assessed together with MMP9 and NFATc1. OVX increased both the extent of F‐actin ring formation and the fluorescence signals for MMP9 and nuclear NFATc1. PU attenuated each of these changes, and the reductions were greater after ZIF‐90@PU treatment (Figure 5C,D). Blank ZIF‐90 again showed little difference from OVX. The combined structural and molecular changes are consistent with impaired formation of the resorptive apparatus and reduced activation of the osteoclast program.

IHC and Western blotting showed the same treatment pattern. NFATc1 and MMP9 staining increased after OVX, decreased with PU, and declined further with ZIF‐90@PU, whereas blank ZIF‐90 produced no clear improvement (Figure 5E,F). Similarly, OVX increased NFATc1, TRAP, CTSK, MMP9, and ATP6V0D2 protein levels; PU lowered these proteins, and ZIF‐90@PU produced the greatest suppression (Figure 5G).

Thus, ZIF‐90@PU reduced osteoclast abundance, disrupted resorption‐associated cytoskeletal structures, and downregulated major osteoclast‐related proteins in vivo.

### 2.6 ZIF‐90@PU Significantly Suppresses Osteoclast Differentiation Through a Lactylation‐Dependent Mechanism

**FIGURE 6:**
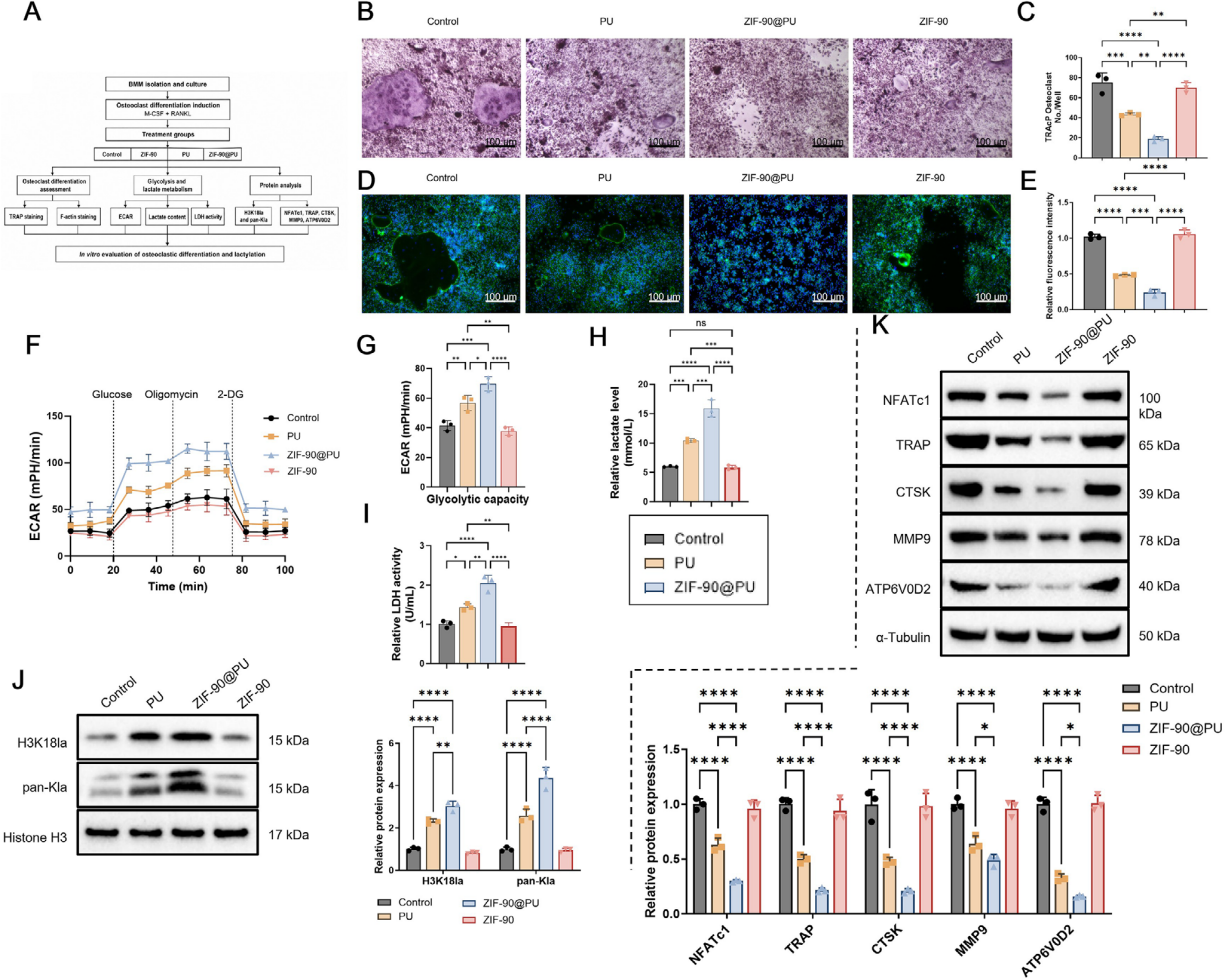
Regulation of osteoclastic differentiation and lactylation in BMMs by ZIF‐90@PU. Note: (A) Schematic diagram illustrating the establishment and evaluation workflow of the BMM osteoclastic differentiation model; (B,C) TRAP staining to assess osteoclastic differentiation; scale bar = 100 µm; (D,E) phalloidin‐based IF staining of F‐actin in BMMs; scale bar = 100 µm; (F,G) Seahorse XF analysis of glycolytic activity (ECAR); (H) lactate content measured by colorimetric assay; (I) LDH activity assay; (J) Western blot analysis of histone lactylation markers (H3K18la and pan‐Kla); (K) Western blot analysis of osteoclast‐associated proteins (NFATc1, TRAP, CTSK, MMP9, and ATP6V0D2). Cell experiments were repeated three times. *p < 0.05, **p < 0.01, ***p < 0.001, ****p < 0.0001. Data were analyzed using one‐way ANOVA with Tukey's HSD post hoc test.

BMMs were differentiated toward osteoclasts to examine the direct cellular effects of ZIF‐90@PU (Figure 6A). PU decreased the number of TRAP‐positive multinucleated cells compared with Control, and ZIF‐90@PU produced a larger decrease; blank ZIF‐90 did not significantly alter TRAP staining (Figure 6B,C). F‐actin staining followed the same pattern, with the strongest disruption of cytoskeletal organization after ZIF‐90@PU treatment (Figure 6D,E).

Metabolic measurements revealed a different treatment pattern. PU increased basal ECAR and the glucose‐stimulated ECAR peak, and both responses were enhanced further by ZIF‐90@PU; blank ZIF‐90 was comparable to Control (Figure 6F,G). Lactate concentration and LDH activity changed in parallel, rising with PU and reaching still higher levels with ZIF‐90@PU (Figure 6H,I).

A carrier‐matched blank ZIF‐90 control was included to determine whether Zn^2+^ released from the framework contributed to these effects. Blank ZIF‐90 did not alter TRAP‐positive cell numbers relative to Control, whereas ZIF‐90@PU markedly reduced osteoclast formation (Figure S5A). Lactate concentrations were likewise similar between Control and blank ZIF‐90 but higher after ZIF‐90@PU treatment (Figure S5B). H3K18la and pan‐Kla were unchanged by blank carrier alone and increased only in the ZIF‐90@PU group (Figure S5C), arguing against blank‐carrier‐derived Zn2+ as the driver of the glycolysis‐lactate‐H3K18la response.

LDHA inhibition was used to test the contribution of lactate generation to histone lactylation. Addition of GSK 2837808A to ZIF‐90@PU‐treated cells reduced lactate and LDH activity toward control values and reversed the increases in H3K18la and pan‐Kla (Figure S6A–C). In the main experiment, PU elevated H3K18la and pan‐Kla, ZIF‐90@PU enhanced these signals further, and blank ZIF‐90 remained similar to Control (Figure 6J). Conversely, NFATc1, TRAP, CTSK, MMP9, and ATP6V0D2 decreased with PU and were suppressed further by ZIF‐90@PU, with no appreciable effect of blank ZIF‐90 (Figure 6K).

These data show that ZIF‐90@PU inhibits osteoclast differentiation while increasing glycolytic activity, lactate production, and histone lactylation. Reversal by the LDHA inhibitor supports a functional contribution of the LDHA‐lactate‐H3K18la axis to the accompanying reduction in the NFATc1‐dependent osteoclastic program.

### 2.7 ZIF‐90@PU Promotes Histone Lactylation and Enhances Osteogenic Differentiation

**FIGURE 7:**
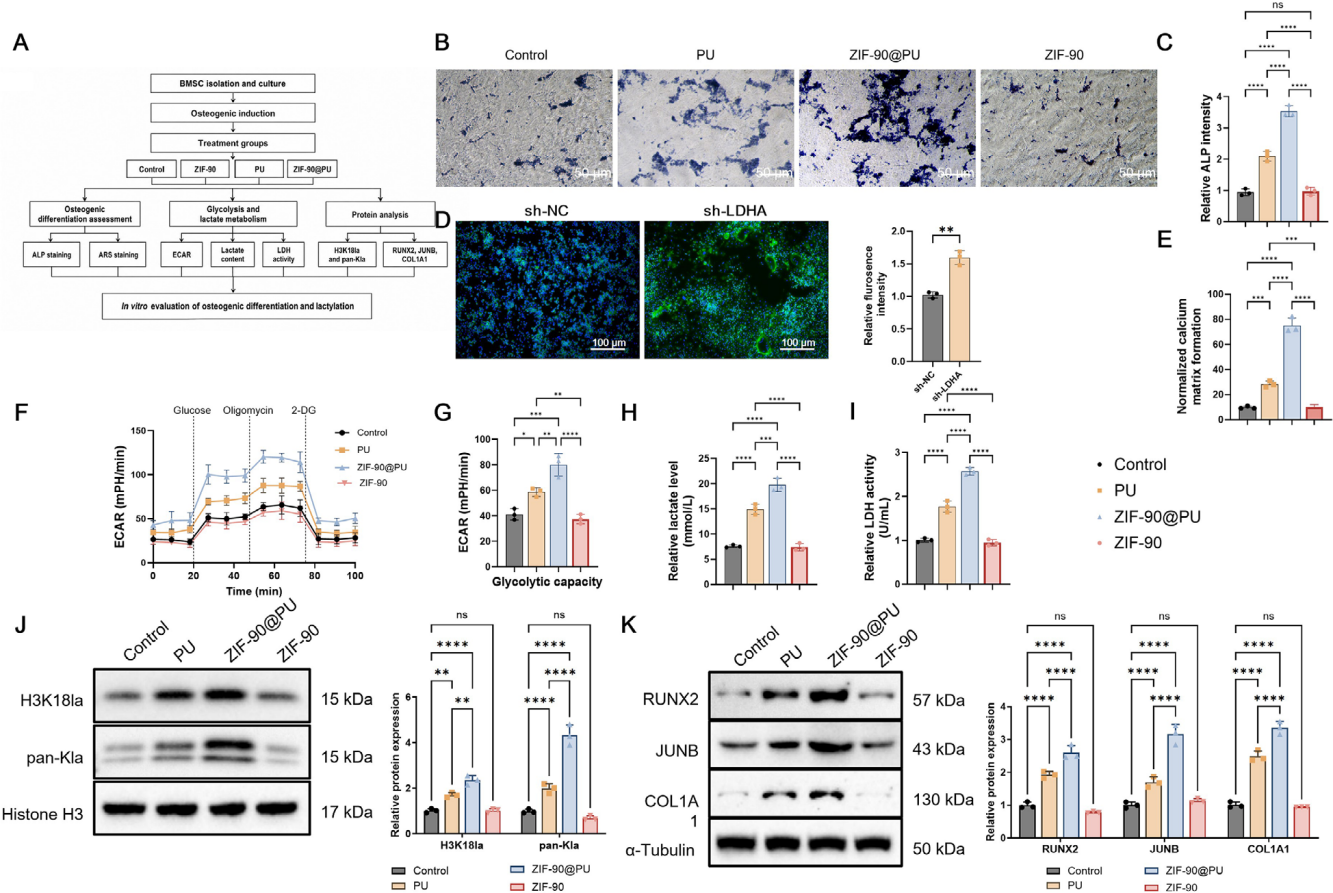
Lactylation‐associated enhancement of BMSC osteogenic differentiation by ZIF‐90@PU. Note: (A) Schematic diagram illustrating the experimental workflow for assessing osteogenic differentiation and lactylation mechanisms in BMSCs; (B,C) ALP staining to evaluate osteogenic differentiation; scale bar = 50 µm; (D,E) ARS staining to assess mineralized nodule formation; scale bar = 50 µm; (F,G) Seahorse analysis measuring ECAR levels to determine glycolytic function; (H) Colorimetric assay quantifying lactate concentration using a lactate detection kit; (I) LDH activity assay to evaluate LDH activity; (J) Western blot analysis of histone lactylation markers H3K18la and pan‐Kla; (K) Western blot analysis of osteogenic markers RUNX2, JUNB, and COL1A1. All experiments were conducted in triplicate. *p < 0.05, **p < 0.01, ***p < 0.001, ****p < 0.0001. Data were analyzed using one‐way ANOVA with Tukey's HSD post hoc test.

BMSCs were subjected to osteogenic induction to determine whether ZIF‐90@PU also affected osteoblast‐lineage differentiation (Figure 7A). PU increased ALP staining relative to Control, and ZIF‐90@PU produced a further increase; blank ZIF‐90 had little effect (Figure 7B,C). ARS staining showed a comparable pattern, with more mineralized nodules after PU and the greatest mineralization after ZIF‐90@PU (Figure 7D,E).

During osteogenic differentiation, PU increased both basal and glucose‐stimulated ECAR, while ZIF‐90@PU enhanced these responses further (Figure 7F,G). Lactate levels and LDH activity increased in the same order across groups (Figure 7H,I). Western blotting showed higher H3K18la and pan‐Kla after PU and an additional increase after ZIF‐90@PU (Figure 7J). RUNX2, JUNB, and COL1A1 also rose with PU and reached higher levels in the ZIF‐90@PU group (Figure 7K).

Accordingly, ZIF‐90@PU promoted the osteogenic phenotype together with increased glycolytic activity, lactate production, H3K18la, and expression of the RUNX2‐JUNB‐COL1A1 osteogenic program.

### 2.8 LDHA Silencing Reverses the ZIF‐90@PU‐Induced, Lactylation‐Mediated Suppression of Osteoclast Activity

Stable LDHA knockdown was established in BMMs and BMSCs to examine the requirement for the glycolysis‐lactate‐histone lactylation pathway (Figure S7A). Both RT‐qPCR and Western blotting confirmed lower LDHA expression in sh‐LDHA cells than in sh‐NC controls (Figure S7B–E). Of the three tested shRNAs, sh‐LDHA‐3 produced the strongest silencing and was therefore used in the subsequent experiments.

**FIGURE 8:**
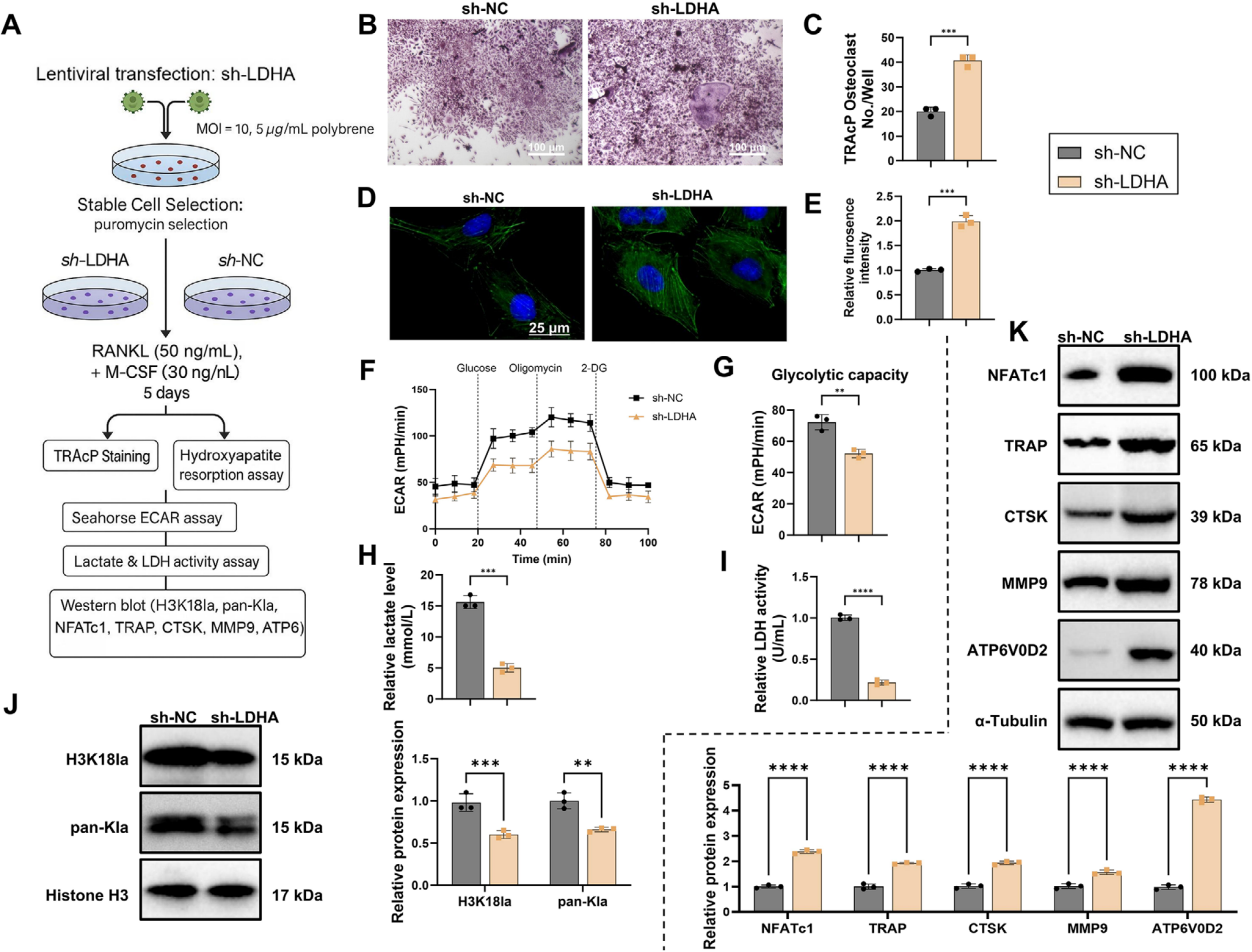
LDHA Silencing attenuates ZIF‐90@PU‐mediated regulation of the glycolysis‐lactylation pathway and osteoclastic differentiation. Note: (A) Schematic diagram showing the construction of stable LDHA‐silenced BMMs and the workflow for osteoclastic differentiation assays; (B,C) TRAP staining to quantify the number of TRAP‐positive osteoclasts in BMMs; scale bar = 100 µm; (D,E) phalloidin‐based IF staining of F‐actin expression in BMMs; scale bar = 25 µm; (F,G) Seahorse analysis of basal ECAR and glucose‐stimulated peak ECAR in BMMs; (H) lactate‐level quantification in BMMs; (I) LDH activity measurement in BMMs; (J) Western blot analysis of histone lactylation markers H3K18la and pan‐Kla in BMMs; (K) Western blot analysis of osteoclastic proteins NFATc1, TRAP, CTSK, MMP9, and ATP6V0D2 in BMMs. All experiments were performed in triplicate. **p < 0.01, ***p < 0.001, ****p < 0.0001.

Under ZIF‐90@PU treatment, LDHA knockdown increased the number of TRAP‐positive multinucleated osteoclasts compared with sh‐NC cells (Figure 8A–C), indicating partial loss of the anti‐osteoclast effect. F‐actin staining was also higher in the sh‐LDHA group (Figure 8D,E), further demonstrating recovery of an osteoclast‐associated cytoskeletal phenotype.

LDHA silencing reduced basal and glucose‐stimulated ECAR (Figure 8F,G), decreased lactate concentration and LDH activity (Figure 8H,I), and lowered H3K18la and pan‐Kla abundance (Figure 8J). In contrast, NFATc1, TRAP, CTSK, MMP9, and ATP6V0D2 were all increased in sh‐LDHA cells relative to sh‐NC cells (Figure 8K).

The coordinated decrease in glycolytic/lactylation indices and recovery of osteoclast‐associated proteins after LDHA knockdown indicate that the anti‐osteoclast activity of ZIF‐90@PU is closely linked to the LDHA‐lactate‐H3K18la pathway.

### 2.9 LDHA Silencing Attenuates ZIF‐90@PU‐Mediated Lactylation and Osteogenic Differentiation

**FIGURE 9:**
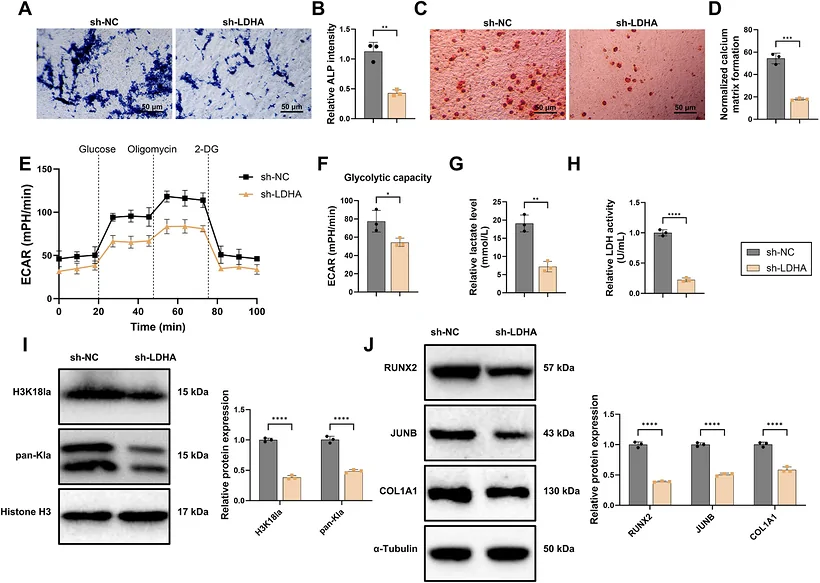
LDHA silencing disrupts ZIF‐90@PU‐induced lactylation and osteogenic differentiation. Note: (A,B) ALP staining for osteogenic differentiation in BMSCs; scale bar = 50 µm; (C,D) ARS staining for mineralized nodule formation in BMSCs; scale bar = 50 µm; (E,F) Seahorse analysis of glycolytic activity (ECAR) in BMSCs; (G) quantitative assessment of lactate production in BMSCs; (H) assessment of LDH activity in BMSCs; (I) Western blot analysis of histone lactylation levels (H3K18la and pan‐Kla) in BMSCs; (J) Western blot analysis of osteogenic markers RUNX2, JUNB, and COL1A1 in BMSCs. All experiments were performed in triplicate. *p < 0.05, **p < 0.01, ***p < 0.001, ****p < 0.0001. Data were analyzed using an independent‐samples *t*‐test.

The effect of LDHA loss on osteogenesis was evaluated in ZIF‐90@PU‐treated BMSCs. Compared with sh‐NC cells, sh‐LDHA cells showed weaker ALP staining (Figure 9A,B) and fewer ARS‐positive mineralized nodules (Figure 9C,D), indicating attenuation of the pro‐osteogenic response.

This phenotypic reduction was accompanied by lower basal and glucose‐stimulated ECAR (Figure 9E,F), decreased lactate concentration and LDH activity (Figure 9G,H), and reduced H3K18la and pan‐Kla levels (Figure 9I). RUNX2, JUNB, and COL1A1 were also downregulated after LDHA silencing (Figure 9J).

Thus, loss of LDHA weakened the ZIF‐90@PU‐associated increases in glycolysis, histone lactylation, and osteogenic marker expression, supporting a role for the LDHA‐lactate‐H3K18la pathway in the osteogenic response.

### 2.10 LDHA Silencing Attenuates the Anti‐Osteoporotic Effects of ZIF‐90@PU In Vivo

**FIGURE 10:**
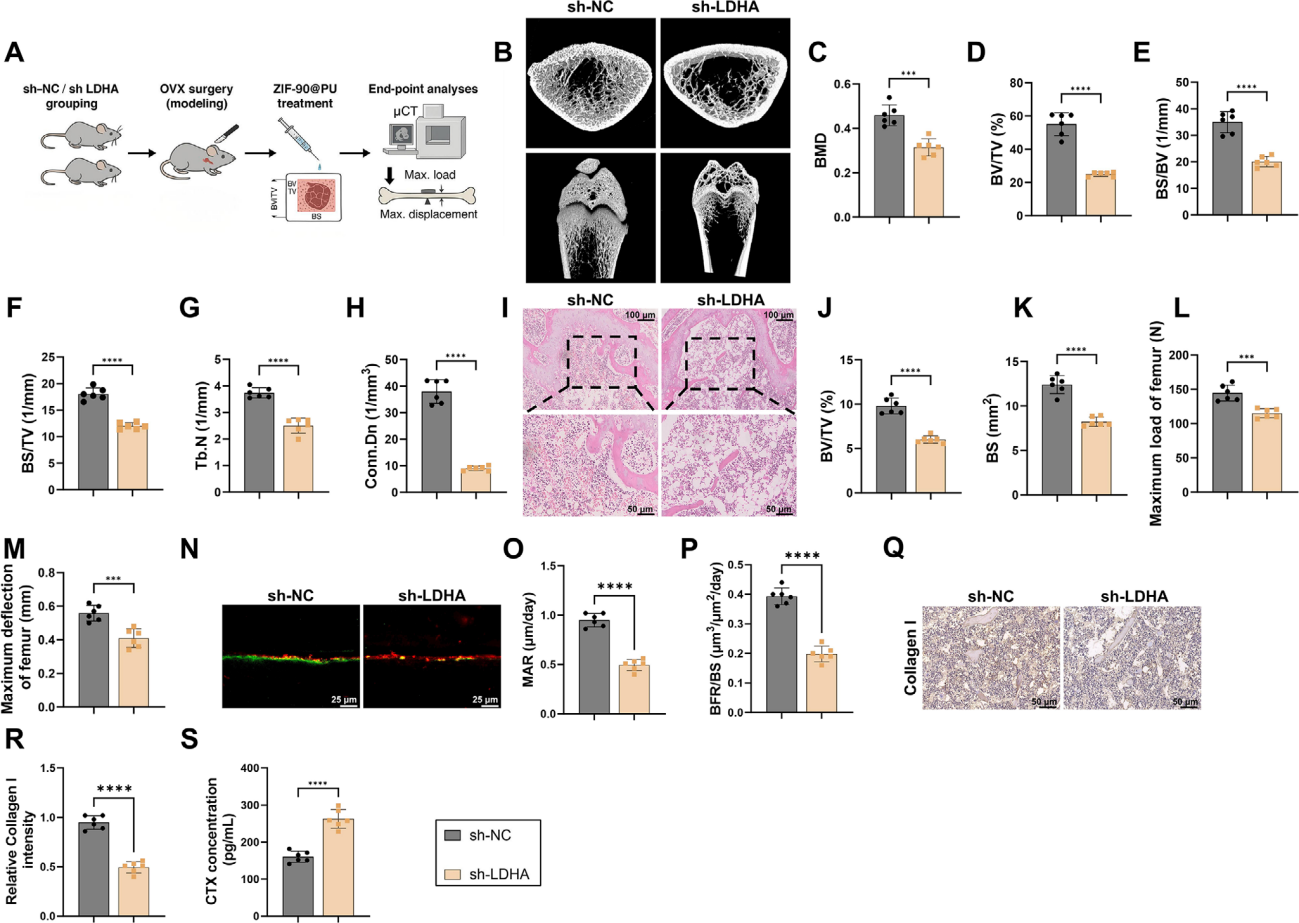
In vivo validation of the mechanism of ZIF‐90@PU in LDHA‐silenced mice with osteoporosis. Note: (A) Schematic overview of the experimental procedure, including lentiviral‐mediated LDHA silencing, OVX modeling, and ZIF‐90@PU treatment; (B) 3D‐µCT reconstruction of trabecular architecture in femoral cancellous bone; (C–H) Quantitative µCT analysis of BMD, BV/TV, BS/BV, BS/TV, Tb.N, and Conn.Dn; (I) H&E staining of femoral histological structure; scale bars = 100 and 50 µm; (J,K) quantitative assessment of BV/TV and BS; (L,M) three‐point bending test to assess femoral maximum load and deflection. (N–P) In vivo double fluorescent labeling with calcein and alizarin red to determine MAR and BFR; (Q,R) IHC staining for collagen I expression in mouse femoral tissue (scale bar = 50 µm); (S) ELISA measurement of serum CTX levels. Each group included 6 animals. **p < 0.01, ***p < 0.001, ****p < 0.0001. Data were analyzed using an independent‐samples t‐test.

The contribution of LDHA was next examined in vivo using sh‐NC‐ and sh‐LDHA‐transduced OVX mice treated with ZIF‐90@PU for six weeks (Figure 10A). µCT showed a more fragmented and sparser trabecular network after LDHA silencing (Figure 10B). BMD, BV/TV, BS/BV, BS/TV, Tb.N, and Conn.Dn were all lower in the sh‐LDHA group than in the sh‐NC group (Figure 10C–H), indicating that systemic LDHA knockdown reduced the structural benefit of ZIF‐90@PU.

H&E staining supported the µCT findings: sh‐LDHA mice displayed wider trabecular spaces, poorer organization, and lower BV/TV and BS (Figure 10I–K). In three‐point bending tests, both maximum load and maximum deflection were also reduced after LDHA silencing (Figure 10L,M), linking the structural deterioration to lower mechanical performance.

Dynamic labeling showed lower MAR and BFR in sh‐LDHA mice than in sh‐NC controls (Figure 10N–P). Type I collagen staining was also reduced after LDHA silencing (Figure 10Q,R), whereas serum CTX increased (Figure 10S), indicating less bone formation together with greater resorptive activity.

Across these readouts, systemic LDHA silencing consistently diminished the skeletal response to ZIF‐90@PU, supporting involvement of the LDHA‐lactate‐H3K18la pathway in its in vivo anti‐osteoporotic activity.

### 2.11 LDHA Silencing Attenuates the In Vivo Anti‐Osteoclastic and Pro‐Osteogenic Effects of ZIF‐90@PU

**FIGURE 11:**
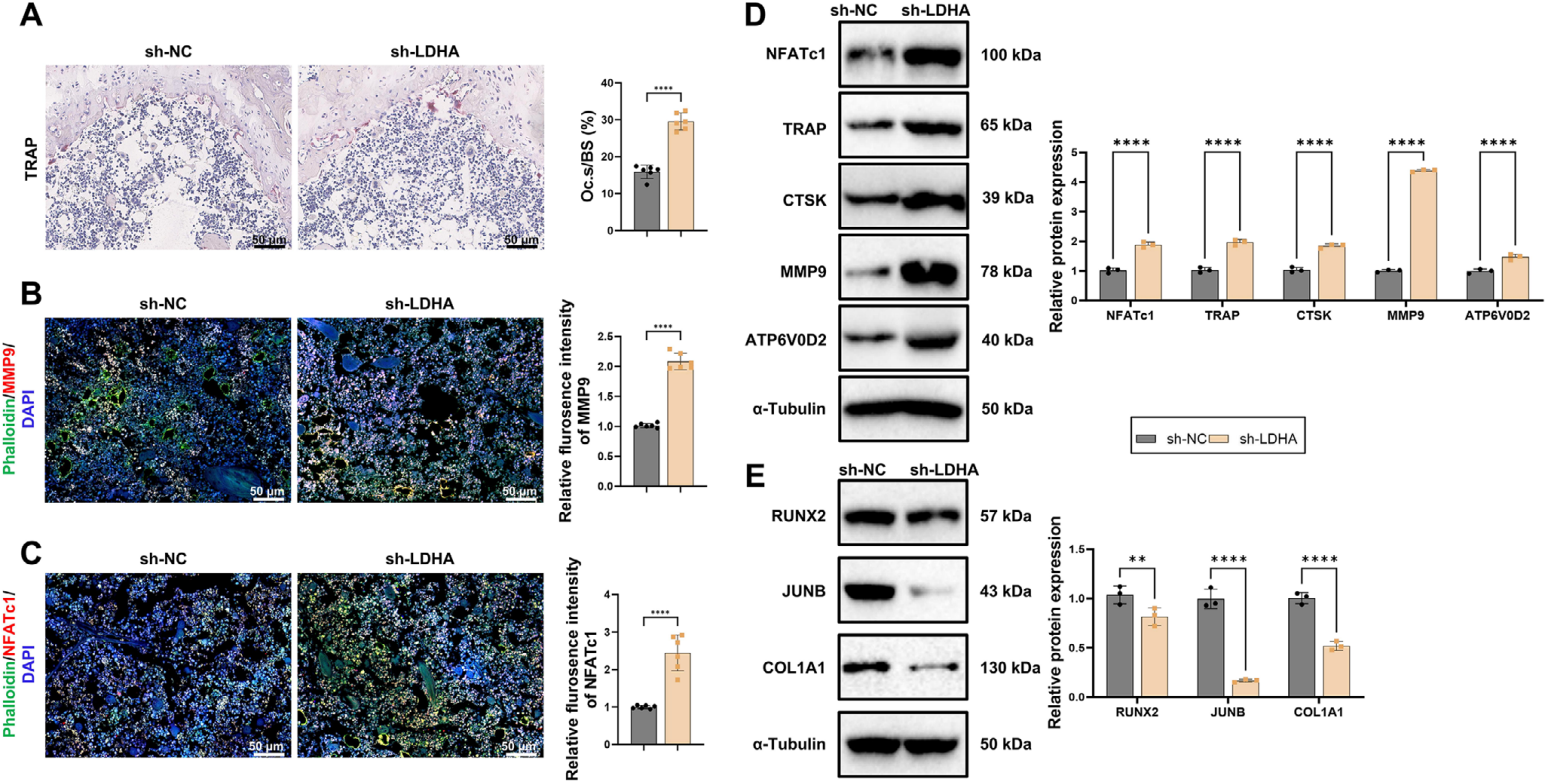
In vivo osteoclastic and osteogenic responses to ZIF‐90@PU after LDHA silencing. Note: (A) TRAP staining to evaluate osteoclast numbers in femoral sections; scale bar = 50 µm; (B,C) IF staining for F‐actin ring formation (phalloidin) and expression of MMP9 and NFATc1; scale bar = 50 µm; (D) Western blot analysis of osteoclast‐associated proteins (NFATc1, TRAP, CTSK, MMP9, ATP6V0D2) in femoral tissues; (E) Western blot analysis of osteoblast‐related proteins (RUNX2, JUNB, COL1A1) in femoral tissues. Each group consisted of 6 animals; the experiments were repeated three times. ****p < 0.0001.

Additional histological and molecular analyses were used to characterize the in vivo osteoclast and osteogenic responses after LDHA silencing. TRAP‐positive multinucleated osteoclasts were more abundant in sh‐LDHA than in sh‐NC bone sections (Figure 11A). F‐actin ring area, MMP9 fluorescence, and nuclear NFATc1 fluorescence were likewise increased in the sh‐LDHA group (Figure 11B,C), indicating attenuation of the anti‐resorptive phenotype associated with ZIF‐90@PU.

Western blotting showed higher NFATc1, TRAP, CTSK, MMP9, and ATP6V0D2 in sh‐LDHA femoral tissue (Figure 11D). In contrast, RUNX2, JUNB, and COL1A1 were reduced after LDHA silencing (Figure 11E). Thus, LDHA loss simultaneously weakened suppression of osteoclast‐related pathways and reduced activation of osteogenic markers.

Together, the in vivo data show that LDHA silencing blunts both the anti‐osteoclastic and pro‐osteogenic responses to ZIF‐90@PU, consistent with participation of the LDHA‐lactate‐H3K18la axis in coordinated regulation of bone remodeling.

## 3 Discussion and Conclusion

The present study developed ZIF‐90@PU as an acid‐responsive puerarin delivery platform and evaluated its effects on osteoporotic bone remodeling together with the associated metabolic‐epigenetic changes. Under acidic conditions, the carrier released PU in a pH‐dependent manner. Across the experimental models, treatment was accompanied by reduced osteoclast activity and enhanced osteogenic differentiation, with coordinated changes in the LDHA‐lactate‐H3K18la pathway. These findings indicate that ZIF‐90@PU can couple microenvironment‐responsive drug release with regulation of bone‐remodeling processes.

Lactate is increasingly recognized not only as a metabolic product but also as a substrate for epigenetic modification. H3K18 lactylation has been linked to changes in chromatin organization and transcriptional control [9, 10], although its contribution to osteoporotic bone remodeling has not been fully established. Here, higher H3K18la levels coincided with attenuation of NFATc1 signaling and lower expression of osteoclast‐associated proteins. In osteogenic cells, the same treatment context was accompanied by increased RUNX2, JUNB, and COL1A1 expression and stronger differentiation. The combined findings therefore place lactylation‐associated regulation at the interface between osteoclastic and osteogenic responses.

Puerarin has previously been reported to restrain osteoclast formation while supporting osteoblast differentiation [14, 15], yet the metabolic basis of these effects remains unclear. In our BMM and BMSC models, PU increased glycolytic activity and lactate production together with H3K18la, and ZIF‐90‐mediated delivery further strengthened these changes. Notably, the rise in glycolytic flux occurred together with suppression of osteoclast differentiation. Although RANKL‐driven glycolysis can provide metabolic support for osteoclastogenesis, lactate also serves as a substrate for histone lactylation. Under the present treatment conditions, increased lactate availability was accompanied by higher H3K18la and attenuation of the NFATc1‐associated osteoclastic program, suggesting that the biological consequence of enhanced glycolysis depends not only on energy production but also on downstream metabolic‐epigenetic utilization of lactate. The balance between these functions may be context dependent and warrants further mechanistic investigation. The micro‐CT data also showed a consistent decrease in BS/BV in the structurally impaired groups: BS/BV was lower after OVX than after sham surgery and was likewise reduced by LDHA silencing under ZIF‐90@PU treatment (Figures 3E and 10E). Although trabecular thinning can increase BS/BV in some osteoporotic settings, the direction of this parameter also depends on the extent of trabecular loss, perforation, and disconnection within the analyzed region. In the present dataset, the lower BS/BV accompanied pronounced reductions in BV/TV, BS/TV, Tb.N, and Conn.Dn, suggesting that extensive loss of trabecular elements contributed to the observed morphometric pattern. Accordingly, BS/BV was interpreted together with the other micro‐CT indices rather than as an isolated measure of osteoporosis severity.

The physicochemical properties of ZIF‐90 make it suitable for stimulus‐responsive drug delivery, particularly because its porous structure supports drug loading and its framework is destabilized under acidic conditions [20, 24]. In the current study, ZIF‐90@PU maintained good biosafety, released PU more readily at lower pH, and showed progressive accumulation in bone tissue over time. These features may improve drug availability within osteoporotic bone without implying active ligand‐mediated targeting. Relative to previously described puerarin formulations, including liposomal, nucleic acid‐framework, and self‐assembled systems, ZIF‐90@PU combines tunable loading with pH‐triggered framework disassembly and a metabolic‐epigenetic response involving the LDHA‐lactate‐H3K18la‐NFATc1‐associated pathway [17, 25, 26].

Single‐cell profiling also provided a descriptive view of changes in bone marrow cellular composition after treatment. The ZIF‐90@PU sample contained a lower proportion of osteoclast‐related cells than the untreated OVX sample. Given the single biological sample analyzed per group, this observation was not used for inferential between‐group statistics. Instead, it was interpreted alongside the independent histological and molecular evidence from TRAP, MMP9, and NFATc1 analyses.

Intervention at the level of LDHA further connected the metabolic changes with the observed bone‐remodeling phenotype. Silencing LDHA reduced the metabolic and lactylation responses induced by ZIF‐90@PU, weakened suppression of osteoclastogenesis, and diminished the enhancement of osteogenic differentiation. The in vivo findings showed the same overall direction, supporting a substantial contribution of LDHA‐dependent metabolism to the activity of the nanoplatform.

Taken together, the multi‐omics and functional experiments outline a coordinated response in which ZIF‐90@PU increased glycolytic flux and lactate availability, raised H3K18la, reduced NFATc1‐associated osteoclastic signaling, and enhanced expression of RUNX2, JUNB, and COL1A1 in osteogenic cells. The platform also combined substantial drug loading with acceptable biosafety, acid‐responsive release, and time‐dependent bone accumulation. Several limitations remain. The OVX model does not fully reproduce clinical osteoporosis, and the scRNA‐seq analysis had limited biological replication. In addition, lentiviral sh‐LDHA was delivered systemically, and LDHA knockdown was not independently quantified in bone marrow‐derived cells or bone tissue in vivo. Tissue‐dependent transduction efficiency and systemic metabolic effects may therefore have contributed to the phenotype observed in the sh‐LDHA group. Future studies incorporating larger single‐cell cohorts, cell‐specific LDHA manipulation, direct in vivo verification of bone‐marrow LDHA suppression, locus‐focused chromatin assays, and longer pharmacokinetic and safety assessments will help refine the proposed mechanism and its translational relevance.

In conclusion, ZIF‐90@PU combined pH‐sensitive PU delivery with suppression of osteoclast activity and enhancement of osteogenic differentiation. These effects were accompanied by coordinated changes in the LDHA‐lactate‐H3K18la pathway, linking metabolic regulation with epigenetic and cellular responses during bone remodeling. The findings support further investigation of acid‐responsive PU nanodelivery as a therapeutic strategy for osteoporosis.

## 4 Materials and Methods

### 4.1 Ethical Statement

This study was conducted in strict accordance with the relevant ethical guidelines and regulations for animal experimentation. All procedures were approved by the Institutional Animal Care and Use Committee (IACUC) (The Animal Ethics Committee of Shanghai Fourth People's Hospital, School of Medicine, Tongji University (TJBH16525401) and KCI Animal Ethics Committee (KCI‐AC20240614‐1)). Animals were housed and cared for in accordance with humane principles, and every effort was made to minimize suffering. At the conclusion of the experiments, all mice were humanely euthanized under ether anesthesia.

### 4.2 Synthesis of ZIF‐90 Nanoparticles

ZIF‐90 was synthesized from separate zinc‐ and ligand‐containing solutions. Zinc nitrate hexahydrate (Zn (NO_3_)_2_∙6H_2_O; 297 mg, 0.996 mmol; 228737, Sigma‐Aldrich, USA) was first dissolved in 16 mL tert‐butanol (t‐BuOH; 99%; PHR2184, Sigma‐Aldrich, USA). A second solution contained imidazole‐2‐carboxaldehyde (2‐ICA; 384 mg, 4.00 mmol; 272 000, Sigma‐Aldrich, USA) and polyvinylpyrrolidone (PVP; 10 mg; average molecular weight 40 000; RDD033, Sigma‐Aldrich, USA) in 16 mL deionized water. After 5 min sonication at 45°C, the aqueous solution was added to the zinc phase while stirring. The precipitate that formed was allowed to react for 5 min and then isolated at 10 000 rpm for 10 min in an Eppendorf 5804R centrifuge (Germany). The collected material was washed six times with 3 mL methanol per wash (99.9%; 34860, Sigma‐Aldrich, USA) before 12 h vacuum drying at room temperature to obtain crystalline ZIF‐90.

### 4.3 Synthesis of ZIF‐90@PU

A one‐pot procedure was used for ZIF‐90@PU formation. Zinc nitrate hexahydrate (297 mg, 0.996 mmol) and PU (8 mg, 0.027 mmol; S2346, Selleck, USA) were combined in 32 mL t‐BuOH/H_2_O (1:1). This mixture was added slowly to 48 mL deionized water containing 2‐ICA (384 mg, 3.996 mmol) and PVP (10 mg). After 5 min of room‐temperature sonication, particles were recovered by centrifugation for 10 min at 10 000 rpm. The pellet was washed thoroughly with methanol and vacuum‐dried for 12 h at room temperature, producing ZIF‐90@PU powder.

Loading was tuned by altering the starting PU‐to‐ Zn (NO_3_)_2_∙6H_2_O feed. Increasing PU from 2 to 16 mg, equivalent to approximate PU/Zn^2+^ molar ratios of 0.007–0.054, produced DL% values of 4.2%–18.7% without appreciable changes in particle morphology or acid responsiveness. The 8 mg condition (DL% = 13.56%) was selected for subsequent studies because it provided a practical compromise between loading capacity and colloidal stability. At higher input, loading approached saturation and particle‐size dispersion became broader [27].

Blank ZIF‐90 was prepared as a carrier‐only control to examine whether Zn^2+^ released from the framework affected osteoclast differentiation. The preparation was identical to that of ZIF‐90@PU except that PU was omitted. SEM, DLS, and PXRD were used to verify that the blank carrier retained morphology, hydrodynamic behavior, and crystallinity comparable to the carrier fraction of ZIF‐90@PU. For BMM experiments, blank ZIF‐90 was dosed on a carrier‐mass basis matched to the ZIF‐90@PU condition. Using the measured DL% of 13.56%, the nanoparticle mass corresponding to 100 µM PU was calculated as PU mass concentration/0.1356. Blank particles were then suspended in PBS at this concentration, sonicated for 5 min, and added to cells. Experiments were repeated independently three times.

### 4.4 Field Emission Scanning Electron Microscopy (FE‐SEM) Analysis

Ultrastructural characterization was carried out with a JEOL JEM‐F200 field‐emission transmission electron microscope (Japan) operated at 200 kV. Nanoparticles were dispersed in anhydrous ethanol and deposited onto ultrathin carbon‐coated copper grids, which were air‐dried before imaging. Crystalline lattice features were inspected by HRTEM. For elemental analysis, HAADF‐STEM imaging was coupled to EDS to determine the spatial distribution of Zn, C, N, and O [28].

Surface morphology was examined by FE‐SEM using a JEOL JSM‐7600F instrument (Japan). Dried nanoparticle powder was spread over conductive adhesive tape on the specimen holder and sputter‐coated with approximately 10 nm of gold using a Quorum Q150R ES system (UK). Images were collected at an accelerating voltage of 5.0 kV and a working distance of 8.0 mm. Both overview and higher‐magnification fields were recorded to assess particle shape and surface features.

### 4.5 Dynamic Light Scattering (DLS) and Zeta Potential Analysis

Hydrodynamic size and zeta potential were determined with a Zetasizer Nano ZS (Malvern Instruments, UK). Before analysis, particle suspensions were diluted eightfold in methanol to 0.125 mg/mL and sonicated at 25°C for 5 min. Size measurements were made in disposable polystyrene cuvettes calibrated for refractive index, with three independent readings per sample and at least 12 scattering accumulations in each run. Surface charge was measured on the same instrument using an electrode cell under a 20 V/cm electric field.

### 4.6 Ultraviolet‐Visible (UV‐Vis) Absorption Spectroscopy

UV‐Vis spectra were obtained on a UV‐2600 spectrophotometer (Shimadzu, Japan) after 30 min instrument warm‐up and methanol baseline correction. Dried ZIF‐90 or ZIF‐90@PU (2.0 mg) was dispersed in 10.0 mL methanol to 0.20 mg/mL, sonicated for 10 min in a water bath, and filtered through a 0.22 µm PVDF membrane (GVWP04700, Merck Millipore, Germany). The clear filtrate was transferred to a 1‐cm quartz cuvette (Hellma Analytics, Germany). Spectra were collected from 100 to 600 nm at 0.5‐nm intervals, with three scans averaged for each sample.

### 4.7 Quantitative Analysis of Drug Loading (DL%)

PU content was determined by UV‐Vis analysis after complete carrier disassembly. Three independent 5‐mg samples of ZIF‐90@PU were incubated for 24 h at room temperature in PBS at pH 5.5 (10010023, Gibco, USA). After centrifugation at 10 000 rpm for 10 min, absorbance of the supernatant was measured at 250 nm and converted to PU concentration from the established calibration relationship. Mean ± SD was calculated from the three determinations. Blank ZIF‐90 subjected to the same procedure was used for background correction. Drug loading and encapsulation efficiency were calculated as follows:

DL(%)=(massofloadeddrug/totalmassofnanoparticles)×100%

EE(%)=(massofloadeddrug/totalmassofdrugadded)×100%

### 4.8 Specific Surface Area and Pore‐Size Analysis (BET/BJH)

Textural properties were characterized by nitrogen adsorption‐desorption on an ASAP 2460 system (Micromeritics, USA). ZIF‐90 and ZIF‐90@PU samples were vacuum‐degassed at 120°C for 12 h before measurement. BET surface area was obtained over P/P0 = 0.05–0.30, and the BJH method was applied to the desorption branch to estimate pore‐size distributions [29].

### 4.9 PH‐Responsive Drug Release Study

pH‐dependent release was assessed in PBS at pH 7.4, 6.5, or 5.5. For each condition, ZIF‐90@PU was suspended at 2 mg/mL and 1 mL was sealed in a dialysis bag with a 3.5‐kDa molecular‐weight cutoff, then immersed in 20 mL of matching buffer in a thermostatic shaker. Samples of the external phase (1 mL) were collected at 0, 2, 4, 8, 12, 24, 48, and 72 h and immediately replaced with equal volumes of fresh PBS. PU in the collected medium was quantified by UV‐Vis spectroscopy to derive cumulative release. Changes in particle size at the three pH values were monitored in parallel by DLS. Each condition was tested in triplicate and summarized using mean values.

### 4.10 Powder X‐Ray Diffraction (PXRD), FTIR, and XPS Analyses

PXRD was used to follow structural changes of ZIF‐90@PU during acid exposure. Measurements were made on an X'Pert Pro‐MPD diffractometer (PANalytical, Netherlands) with Cu Kα radiation (λ = 1.54178 Å) at 40 kV and 40 mA. Dried ZIF‐90@PU (20 mg per condition) was incubated at 10 mg/mL in PBS adjusted to pH 7.4, 6.5, 6.0, 5.5, 5.0, or 4.5 for 24 h at 25°C without agitation. Material was then pelleted at 12 000 rpm for 10 min, washed once with anhydrous ethanol (≥99.8%; PHR1070, Sigma‐Aldrich, USA), and vacuum‐dried for 12 h at 40°C. Finely ground samples were placed on low‐background holders and scanned continuously from 2θ = 5° to 30° using 0.02° steps and 0.5 s per step. The divergence slit was 1/4°, the receiving slit 0.04 rad, and the stage rotation 15 rpm; identical acquisition settings were used for all specimens. The 2θ zero point was calibrated with NIST SRM 640d silicon. HighScore Plus (PANalytical) was used for background correction, Kα2 removal, vertical offsetting, and comparison with a simulated Cu Kα ZIF‐90 pattern. FTIR was performed separately on PU, ZIF‐90, and ZIF‐90@PU over 4000–400 cm‐1, with attention to O─H (3200–3500 /cm), C═O/C═N (1650–1700 /cm), C═C/C═N (1600–1500 /cm), C─O─C/C─O (1260–1000 /cm), and Zn─N (420–450 /cm) bands. XPS measurements used Al Kα excitation on dried ZIF‐90 and ZIF‐90@PU. Survey scans and high‐resolution C 1s, O 1s, N 1s, and Zn 2p spectra were acquired; binding energies were referenced to C 1s at 284.8 eV, and peak fitting was completed after background subtraction.

### 4.11 Isolation, Culture, and Grouping of Bone Marrow‐Derived Macrophages (BMMs) and Bone Marrow‐Derived Mesenchymal Stem Cells (BMSCs)

Primary BMMs were derived from femoral and tibial marrow of 6–8‐week‐old C57BL/6J mice (20–22 g; No. 219, Vital River, USA). Under sterile conditions, attached soft tissue was removed and the marrow cavities were repeatedly flushed with sterile PBS. Cell suspensions were passed through a 70 µm strainer, treated for 5 min at room temperature with red blood cell lysis buffer (C3702, Beyotime, China), and centrifuged at 300 × *g* for 5 min. Pellets were resuspended in α‐MEM (12571‐063, Gibco, USA) containing 10% FBS (F8687, Gibco, USA), 1% penicillin‐streptomycin (TMS‐AB2, Gibco, USA), and recombinant mouse M‐CSF (30 ng/mL; 315‐02‐10UG, PeproTech, USA). After 24 h at 37°C and 5% CO2, non‐adherent cells were removed and fresh M‐CSF‐containing medium was supplied. Adherent cells were maintained for a further 2–3 days before osteoclast‐induction experiments.

BMSCs were isolated from femoral and tibial marrow of 6–8‐week‐old C57BL/6 mice. Marrow recovered by PBS flushing was filtered through a 70 µm strainer and centrifuged for 5 min at 300 × *g*. The pellet was resuspended in low‐glucose DMEM (SNM‐002A, Sunncell, China) supplemented with 10% FBS and 1% penicillin‐streptomycin and cultured at 37°C in 5% CO2. Non‐adherent cells were discarded after 24 h, fresh medium was added, and cultures were thereafter fed every 2–3 days. Cells were passaged at approximately 80% confluence, and passages 2–4 were used for experiments.

For osteoclastogenesis, BMMs were maintained for 5 days in M‐CSF (30 ng/mL; 315‐02‐10UG, PeproTech, USA) plus RANKL (50 ng/mL; 315‐11C‐10UG, PeproTech, USA), with cytokine‐containing medium renewed every 2 days. Osteogenic induction of BMSCs began at approximately 80% confluence using EMEM supplemented with 10% FBS, 10 mM β‐glycerophosphate (G9422, Sigma‐Aldrich, USA), 50 µg/mL ascorbic acid (AX1775, Sigma‐Aldrich, USA), and 100 nM dexamethasone (D4902, Sigma‐Aldrich, USA). Osteogenic medium was replaced at 2‐day intervals [30].

Cell‐based treatment groups comprised PBS control, PU (100 µM), blank ZIF‐90 at the carrier mass corresponding to the ZIF‐90@PU condition, and ZIF‐90@PU providing 100 µM PU. For LDHA‐silencing experiments, negative‐control lentivirus‐infected cells (sh‐NC) and LDHA‐silenced cells (sh‐LDHA) both received ZIF‐90@PU at 100 µM PU equivalent. Treatments lasted 24 h, and all experiments were independently repeated three times.

### 4.12 LDHA Inhibitor Treatment

BMMs undergoing osteoclast induction were used to assess the effect of pharmacological LDHA inhibition. GSK 2837808A (20 µM; S7631, Selleck, USA) was applied to three conditions: Control (0.1% DMSO), ZIF‐90@PU, and ZIF‐90@PU plus GSK 2837808A. Fresh inhibitor was supplied whenever the differentiation medium was renewed. At the induction endpoint, cells were harvested for lactate, LDH activity, histone lactylation, and osteoclast‐marker analyses. Three independent experiments were completed [31].

### 4.13 Lentiviral Transduction

Stable LDHA knockdown in BMMs and BMSCs was generated by lentiviral transduction. Virus was produced by Sangon Biotech (Shanghai, China) using pSuper‐retro‐puro constructs together with gag/pol and VSVG packaging plasmids (#113535, #14887, and #8454; Addgene, USA). These plasmids were introduced into HEK293T cells (SNL‐015, Sunncell, China) with Lipofectamine 2000 (11668030, Thermo Fisher, USA). Supernatant collected 48 h later was filtered through 0.45 µm membrane, concentrated by centrifugation, and titrated. Target cells at roughly 75% confluence were exposed to virus at MOI 10 (working titer ∼5 × 10^6 TU/mL) in 5 µg/mL polybrene (TR‐1003, Merck, USA). After 4 h, an equal volume of fresh medium was added; complete medium replacement followed at 24 h. Stable populations were selected initially with 2 µg/mL puromycin (E607054, Sangon Biotech, China), after which puromycin was increased stepwise to 4, 6, 8, and 10 µg/mL during passaging. Knockdown was evaluated by WB and RT‐qPCR, and the most effective construct was carried forward. shRNA sequences are provided in Table S1.

### 4.14 Cy5.5 Labeling and Cellular Uptake Assay

Cy5.5 labeling was performed with Cy5.5 NHS ester (R‐C‐3583, Xi'an Ruixi Biological Technology Co., Ltd., China). After conjugation, free dye was removed and the nanoparticles were washed three times in PBS. BMSCs and BMMs grown for 24 h on glass‐bottom dishes were then exposed to Cy5.5‐ZIF‐90@PU at 50 µg/mL. Cells were collected at 0, 1, 2, and 4 h. Before imaging, cultures were washed three times with PBS to eliminate extracellular particles, fixed for 10 min in 4% paraformaldehyde (158127, Sigma‐Aldrich, USA), and counterstained with DAPI (C1005, Beyotime, China) for 15 min at room temperature. Intracellular fluorescence was visualized on a Leica TCS SP8 confocal microscope (Germany). Parallel samples were resuspended in 500 µL PBS and analyzed on a BD FACSAria III flow cytometer (USA) to quantify particle uptake.

### 4.15 In Vitro Biosafety Evaluation

Cytotoxicity was first examined by CCK‐8 assay (C0041, Beyotime, China). BMSCs and BMMs were plated in 96‐well plates and exposed for 24 h to ZIF‐90@PU at 50, 100, 150, 200, or 400 µM in complete medium containing 10% FBS and 1% penicillin‐streptomycin. CCK‐8 reagent (10 µL) was then added to each well for a further 2 h, after which absorbance was recorded at 450 nm with a BioTek Synergy H1 reader (USA). Three independent experiments were performed, with five replicate wells at each concentration, and viability was calculated from the absorbance values.

Cell survival was also evaluated with a calcein AM/PI live‐dead staining kit (PF00007, Proteintech, USA). BMSCs and BMMs were cultured separately in 24‐well plates for 24 h in the presence of 100 µM ZIF‐90@PU, with matched untreated controls. Cultures were rinsed two to three times in PBS and incubated in darkness for 15–20 min at room temperature with 2 µM calcein AM and 4.5 µM PI. Fluorescence‐microscopy samples were imaged immediately. For flow cytometry, stained cells were analyzed within 30 min on a BD FACSCanto II instrument (BD Biosciences, USA); FlowJo v10 (Tree Star, USA) was used for data processing. Calcein AM and PI signals were recorded at 530 and 617 nm, respectively.

### 4.16 Hemolysis Assay

Hemocompatibility was tested by incubating mouse erythrocytes with ZIF‐90@PU. Nanoparticle preparations were tested at 50, 100, 150, 200, and 400 µM, with PBS and deionized water serving as negative and positive controls. Fresh anticoagulated blood from C57BL/6 mice was diluted in PBS (pH 7.4) to obtain a 2% RBC suspension. Equal volumes of RBC suspension and test solution were incubated for 1 h at 37°C. Intact cells were removed by centrifugation at 2000 × *g* for 10 min, and absorbance of the supernatant was measured at 540 nm to calculate the hemolysis rate.

### 4.17 Multicolor Immunofluorescence Co‐Localization in Bone Tissue

Bone‐tissue co‐localization was evaluated 48 h after intravenous administration of DiR‐labeled ZIF‐90@PU to OVX mice. Femora were fixed, decalcified, paraffin‐embedded, and cut into 5 µm sections. Following citrate‐buffer antigen retrieval (pH 6.0) and 30 min blocking with 5% BSA, sections were incubated overnight at 4°C with anti‐F4/80 (ab6640, Abcam, UK) and anti‐TRAP (PA5‐116970, Thermo Fisher, USA). Alexa Fluor 594 donkey anti‐rat IgG (A‐21209, Thermo Fisher, USA) and Alexa Fluor 647 donkey anti‐rabbit IgG (A‐31573, Thermo Fisher, USA) were then applied for 1 h at room temperature in darkness. DAPI counterstaining lasted 5 min. DiR was excited at 633 nm, and fluorescence images were collected on an LSM 880 confocal microscope (Zeiss, Germany) [32].

### 4.18 Seahorse Extracellular Acidification Rate (ECAR) Assay

Glycolytic function was quantified as extracellular acidification rate with the Seahorse XF Glycolysis Stress Test Kit (103020‐100, Agilent Technologies, USA). Cells were seeded at 2 × 10^4 per well in XF24 plates. After the designated treatment period, treatment medium was removed and replaced with fresh glucose‐free Seahorse assay medium. The assay then proceeded with sequential injections of glucose (10 mM), oligomycin (1 µM), and 2‐deoxy‐D‐glucose (2‐DG; 50 mM). ECAR was monitored in real time on a Seahorse XF24 Analyzer and processed using Wave v2.6.1 software (Agilent Technologies, USA).

### 4.19 Lactate Assay

Extracellular lactate was measured using the CB11296‐Mu colorimetric kit (COIBO BIO, China). Culture supernatants were collected from each condition and processed with the kit reagents. Absorbance was read at 530 nm, and lactate concentrations were calculated and reported in mmol/L.

### 4.20 Lactate Dehydrogenase (LDH) Activity Assay

LDH activity in cell lysates was determined with kit CB10587‐Mu (COIBO BIO, China). Lysates were prepared for the assay, absorbance was measured at 450 nm on a microplate reader, and enzyme activity was expressed as U/mL protein.

### 4.21 RT‐qPCR Analysis of Gene Expression

Total RNA from cells or tissues was isolated with TRIzol reagent (A33254, Thermo Fisher, USA) and converted to cDNA using RR047A reverse‐transcription reagents (Takara, Japan). qPCR reactions were prepared with SYBR Premix Ex Taq II (DRR081, Takara, Japan) and run on an ABI7500 real‐time system (Thermo Fisher, USA). Cycling consisted of 95°C for 30 s, then 40 cycles of 95°C for 5 s and 60°C for 30 s, followed by melt‐curve acquisition at 95°C for 15 s, 60°C for 60 s, and 90°C for 15 s. Gapdh served as the reference gene. Each biological experiment was repeated three times, with reactions run in triplicate wells. Relative transcript abundance was determined by the 2^‐ΔΔCt approach, where ΔCt = Ct(target)—Ct(reference) and ΔΔCt = ΔCt(experimental)—ΔCt(control). Primer sequences are listed in Table S2.

### 4.22 Construction and Grouping of an Osteoporosis Mouse Model

Female C57BL/6J mice aged 11 weeks (20‐22 g; No. 219, Vital River, USA) were used for osteoporosis modeling. Animals were maintained under SPF conditions at 22 ± 2°C, 50 ± 10% relative humidity, and a 12‐h light/dark cycle with unrestricted chow and water. Bilateral ovariectomy was carried out under 1%–2% isoflurane anesthesia (R510‐22‐10, RWD, China); sham‐operated animals underwent ovarian exposure without removal. To reduce postoperative infection risk, 0.2% chloramphenicol was provided in drinking water for 3 days. Standard therapeutic intervention began 7 days after surgery. For the LDHA‐silencing arm, mice first received sh‐LDHA or sh‐NC lentivirus (GeneChem, Shanghai, China; 1 × 10^8 TU/mouse) through the tail vein. OVX was performed 2 weeks later, followed by 7 days of postoperative recovery before treatment.

The principal in vivo study included six groups (n = 6/group): Sham (PBS), OVX (PBS), PU (100 mg/kg), ZIF‐90@PU at a PU‐equivalent dose of 100 mg/kg, sh‐NC plus ZIF‐90@PU (PU equivalent 100 mg/kg), and sh‐LDHA plus ZIF‐90@PU (PU equivalent 100 mg/kg). A separate blank ZIF‐90 carrier group was included in the in vivo osteoclast analyses presented in Figure 5. Beginning on postoperative day 7, tail‐vein treatments were administered every 3 days for 6 weeks. ZIF‐90@PU was suspended in PBS, and PBS‐treated groups received matched injection volumes.

At the endpoint, femora were harvested following euthanasia and scanned by high‐resolution 3D‐µCT on a SkyScan 1176 system (Bruker, Belgium). Acquisition used 9 µm slices, 80 kV, 100 µA, and 300 ms exposure. CTAn software (Bruker micro‐CT, Belgium) was used for reconstruction and quantification of BMD, BV/TV, BS/BV, BS/TV, Tb.N, and Conn.Dn. Group means entered the statistical analysis [33].

### 4.23 In Vivo Distribution of Nanoparticles

For fluorescence tracing, lyophilized ZIF‐90@PU and DiR (HY‐D1048, MedChemExpress, USA) were each prepared in anhydrous ethanol at 1 mg/mL. One milliliter of nanoparticle suspension was combined with 50 µL DiR and magnetically stirred for 2 h at room temperature in the dark. The mixture was centrifuged at 8000 rpm for 10 min, after which the pellet was washed sequentially three times with anhydrous ethanol and deionized water to remove free dye. Labeled nanoparticles were resuspended in PBS and kept at 4°C protected from light.

OVX mice received 0.2 mL of DiR‐labeled ZIF‐90@PU by tail‐vein injection. Animals were euthanized at 4, 12, 24, or 48 h (*n* = 3/time point), and the heart, liver, spleen, lungs, kidneys, femurs, and tibias were excised, rinsed with PBS, and imaged on an IVIS Lumina III platform (PerkinElmer, USA). Signals were expressed as average radiant efficiency ([p/s/cm^2^/sr]/[µW/cm^2^]). Changes in fluorescence over time were used to calculate the degradation rate of DiR‐ZIF‐90@PU. Free DiR‐PU served as a 48‐h comparator [34].

### 4.24 In Vivo Calcein and ARS Double Fluorescence Labeling

Dynamic bone formation was assessed by sequential calcein and ARS labeling. Calcein (20 mg/kg; C0875, Sigma‐Aldrich, USA) was given intraperitoneally 14 days before euthanasia, and ARS (40 mg/kg; A5533, Sigma‐Aldrich, USA) was administered 7 days later, yielding a 7‐day interval between labels. Mice were euthanized 48 h after the ARS injection. Tibiae were collected immediately, fixed in 4% paraformaldehyde, dehydrated through graded ethanol, and embedded undecalcified in PMMA. Transverse hard‐tissue sections were prepared from the tibial mid‐diaphysis without additional staining.

Confocal microscopy was used to record calcein and ARS fluorescence sequentially at excitation wavelengths of 488 and 561 nm, respectively. MAR was derived from the mean distance separating the two fluorescent fronts. BFR was calculated as MAR × MS/BS, where MS denotes mineralizing surface and BS denotes bone surface.

### 4.25 ELISA Measurement of Serum C‐Terminal Telopeptide of Type I Collagen (CTX) in Mice

At the animal‐study endpoint, mice were fasted for 12 h before retro‐orbital blood collection. Blood was transferred to tubes without anticoagulant, allowed to stand for 30 min at 22°C (room temperature), and centrifuged at 3000 rpm (4°C for 10 min). Serum was stored at −80°C until analysis. CTX was measured with the EEL219 ELISA kit (Thermo Fisher Scientific, USA). Samples and standards were added to antibody‐coated wells and processed through the required wash, enzyme‐conjugate, and chromogenic steps. Absorbance was recorded at 450 nm after reaction termination, and CTX concentrations were interpolated from the standard curve. Duplicate wells were used for every sample.

### 4.26 Bone Marrow Cell Subset Distribution Analysis

Cellular distribution of DiR‐ZIF‐90@PU within bone marrow was analyzed 48 h after tail‐vein injection into OVX mice. After erythrocyte lysis, staining with CD11b, Ly6G, and CD31 antibodies was used to define monocyte/macrophage‐lineage cells, neutrophils, and endothelial cells. DiR mean fluorescence intensity was calculated for each gated population in FlowJo v10.

### 4.27 Statistical Analysis

Except for the descriptive scRNA‐seq comparison, which contained one biological sample in each group, quantitative data were obtained from at least three independent experiments and are reported as mean ± SD. Two‐group comparisons used independent‐samples *t*‐tests. When three or more groups were compared, one‐way ANOVA with Tukey's HSD post hoc testing was applied when parametric assumptions were satisfied. Mann‐Whitney U and Kruskal‐Wallis H tests were used for nonparametric two‐group and multi‐group comparisons, respectively, when normality or variance assumptions were not met. Statistical analyses were completed in GraphPad Prism 9.5.0 and R 4.2.1. All tests were two‐tailed, with *p <* 0.05 considered significant and p ≥ 0.05 considered nonsignificant.

## Author Contributions

J.J.L. and X.J.S. conceived and designed the study. J.J.L., Y.X., and S.W. performed the experiments, J.J.L, S.W., and J.J.Y analyzed the data. J.J.L. and Y.X. wrote the manuscript. Y.C. and X.J.S. reviewed the manuscript. During the revision stage, **Yue Xi**, **Sheng Wang** and **Ye Chen** supplemented most of the experiments and data, as well as revised and polished the manuscript.

## Funding

This work was supported by National Natural Science Foundation of China (NSFC) Youth Fund Project (No. 82502887), “Medicine +X” interdisciplinary research program of Tongji University (No. 2025‐0674‐YB‐04), the Clinical Key Specialty Construction Project of Hongkou District Health Commission (No. HKLCZD2024B02), Mandatory project of Nantong Health Commission (No. MS2025113), and Scientific Research Project of Jiangsu Geriatrics Society (No. JGS2026MS01).

## Ethics Statement

This study was conducted in strict accordance with the relevant ethical guidelines and regulations for animal experimentation. All procedures were approved by the Institutional Animal Care and Use Committee: The Animal Ethics Committee of Shanghai Fourth People's Hospital, School of Medicine, Tongji University (TJBH16525401) and KCI Animal Ethics Committee (KCI‐AC20240614‐1). All animals were housed and cared for in accordance with humane principles, and every effort was made to minimize suffering.

## Conflicts of Interest

The authors declare no conflicts of interest.

## Supporting information




**Supporting File 1**: advs77869‐sup‐0001‐SuppMat.docx.

**Supporting File 2**: advs77869‐sup‐0002‐Figures.zip.

## Data Availability

The data that support the findings of this study are available from the corresponding author upon reasonable request.
