# Supplementary material for: Puerarin‑Loaded pH‑Responsive ZIF‑90 Nanoparticles Ameliorate Osteoporosis via Enhancing H3K18 Lactylation and Suppressing Aberrant Osteoclast Activation
Source: Adv Sci (Weinh). 2026 Sep 27:e77869. Online ahead of print. doi: 10.1002/advs.77869 (PMC13616396; doi:10.1002/advs.77869)
Supplement: Supplementary file 1 [file ADVS-9999-e77869-s002.docx]

**Supplementary materials (**Experiments and tables**)**

1. Supplementary Experiments

**1.1. scRNA-seq Library Construction and Sequencing**

Single-cell suspensions were adjusted after counting to 700-1200 cells/μL. Cell partitioning and cDNA generation were performed on the Chromium Controller using the Chromium Single Cell 3′ Library & Gel Bead Kit v3 (10x Genomics, USA). Libraries were produced through the standard reverse-transcription, amplification, and construction workflow. Quality was checked on an Agilent 2100 Bioanalyzer and concentration on a Qubit 4.0 fluorometer (Thermo Fisher Scientific, USA). Sequencing was carried out on an Illumina NovaSeq 6000 with PE150 reads at an average depth of at least 50,000 reads/cell.

**1.2 Sequencing Data Processing and Analysis**

Cell Ranger v6.1.2 (10x Genomics, USA) was used to process raw scRNA-seq reads against the mm10 mouse reference genome (Ensembl release 104). The pipeline included FASTQ quality control, alignment, gene counting, and identification of cell barcodes. Cells with <200 or >6000 detected genes or with mitochondrial transcripts >10% were removed before downstream analysis. The filtered gene-by-cell matrix was retained for subsequent processing.

Downstream single-cell analysis was performed in Seurat v4.3.0. Data were normalized with NormalizeData, and the 2000 most variable genes were selected for PCA. UMAP was used for nonlinear dimensionality reduction, and FindClusters with a resolution of 0.8 was used to define cell clusters. Cell identities were assigned from established lineage-marker expression with reference to the CellMarker database (http://bio-bigdata.hrbmu.edu.cn/CellMarker/).

**1.3 Sample Collection, RNA Library Construction, and High-Throughput Sequencing**

For bulk RNA-seq, three PBS-treated control osteoclast samples and three ZIF-90@PU-treated samples were collected after 24 h exposure (PU equivalent, 100 μM). Cells were rinsed three times with PBS and lysed in TRIzol (15596026CN, Invitrogen, USA) for total RNA isolation. NanoDrop 2000 (Thermo Fisher Scientific, USA) was used to measure concentration and purity, and an Agilent 2100 Bioanalyzer was used to determine RNA integrity. Samples with RIN ≥ 7 proceeded to library preparation.

mRNA-enriched libraries were constructed with the NEBNext Ultra II RNA Library Prep Kit (E7770L, New England Biolabs, USA), including fragmentation, cDNA synthesis, end repair, and adapter ligation. Library integrity was confirmed by Agilent 2100 Bioanalyzer and concentration determined with Qubit 4.0 fluorometry (Thermo Fisher Scientific, USA). PE150 sequencing was performed on an Illumina NovaSeq 6000, generating at least 6 GB of sequence data for each sample.

1.4 **RNA-seq Data Processing and Analysis**

Bulk RNA-seq data were cleaned with fastp v0.23.2. Remaining reads were aligned to GRCm39 (Ensembl release 104) with STAR v2.7.10a. Gene-level counts were generated by featureCounts v2.0.3 and normalized for reporting as FPKM.

Differential-expression testing was conducted in R v4.2.2 using limma v3.54.0, with |log2FC| > 1 and *p <* 0.05 defining DEGs. EnhancedVolcano v1.16.0 was used for volcano plots. Gene Ontology and KEGG enrichment analyses were performed in ClusterProfiler v4.6.2. GO terms were considered across BP, CC, and MF categories; KEGG annotations were obtained from https://www.kegg.jp/. Enrichment results meeting *p <* 0.05 were visualized as bubble plots in ggplot2 v3.4.1.

1.5 **Tartrate-Resistant Acid Phosphatase (TRAP) Staining**

TRAP activity was used to quantify osteoclast differentiation. Cells were washed three times with PBS, fixed in 4% paraformaldehyde for 15 min, and stained with the MK301 TRAP kit (Takara, Japan). Multinucleated TRAP-positive cells containing at least three nuclei were identified microscopically (Leica DM5000B, Germany) and counted on a per-well basis.

1.6 **Immunofluorescence (IF) Staining**

For cellular F-actin staining, ZIF-90@PU-treated cells (100 μM) were fixed for 20 min with 4% paraformaldehyde, permeabilized for 10 min in 0.1% Triton X-100, and blocked for 30 min with 1% BSA. Phalloidin-iFluor 647 (ab176759, Abcam, UK) was applied for 30 min at room temperature in darkness, followed by a 5-min DAPI counterstain (1 μg/mL).

For femoral sections, antigen retrieval was performed in citrate buffer (pH 6.0; C2488, Sigma-Aldrich, USA) using a pressure cooker, followed by 30 min blocking in 5% BSA. Primary antibodies to MMP9 (1:200; MA5-15886, Thermo Fisher, USA) and NFATc1 (1:200; PA5-90432, Thermo Fisher, USA) were applied overnight at 4°C. Sections then received 594-conjugated secondary antibody (1:500; A-11012, Thermo Fisher, USA) for 1 h at room temperature in darkness. F-actin was visualized with Phalloidin-iFluor 488 (ab176753, Abcam, UK) and nuclei with DAPI (1 μg/mL). Imaging was performed on an LSM 880 confocal microscope (Zeiss, Germany).

1.7 **Alkaline Phosphatase (ALP) Staining**

Osteogenic ALP activity was visualized using kit C3206 (Beyotime, China). Cells were seeded at 2 × 10^4 per well in 24-well plates. After treatment, cultures were rinsed with PBS, fixed in 4% paraformaldehyde for 15 min, and incubated with ALP chromogenic substrate for 30 min at room temperature. Stained cultures were imaged by light microscopy after washing, and ImageJ was used to quantify staining intensity.

1.8 **Alizarin Red S (ARS) Staining**

Mineral deposition was assessed with an Alizarin Red S kit (G1450, Solarbio, China). Following PBS washing, cells were fixed in 4% paraformaldehyde for 15 min and stained for 20 min at room temperature with 1% ARS adjusted to pH 4.2. Unbound stain was removed with deionized water before light-microscopy imaging. For semiquantitative analysis, bound ARS was dissolved in 1 mL 0.1 M NaOH and absorbance was measured at 480 nm.

1.9 **Western Blot Analysis**

Proteins were extracted from cells or tissues in RIPA buffer containing 1% PMSF (P0013B, Beyotime, China). After clarification, protein concentration was measured with the BCA kit (P0011, Beyotime, China), and lysates were adjusted to 1 μg/μL. Aliquots of 100 μL were heated for 10 min at 100°C and stored at -80°C until analysis. According to target molecular weight, 8%-12% SDS-PAGE gels were used. Each lane received 50 μg protein; electrophoresis was run first at 80 V and then at 120 V for a total of 2 h. Proteins were transferred to PVDF membranes (1620177, Bio-Rad, USA) by wet transfer at 250 mA for 90 min.

Membranes were blocked for 1 h at room temperature in 5% non-fat milk prepared in 1×TBST and then washed for 10 min. Primary antibodies listed in Table S3 were incubated with membranes overnight at 4°C. After three 10-min TBST washes, HRP-conjugated goat anti-rabbit IgG (ab6721, 1:5000; Abcam, UK) or goat anti-mouse IgG (ab6789, 1:5000; Abcam, UK) was applied for 1 h at room temperature. Membranes were subsequently washed three times for 5 min and developed with ECL substrate (1705062, Bio-Rad, USA) for 1 min. Chemiluminescence was recorded on an ImageQuant LAS 4000C system (GE, USA). α-Tubulin or GAPDH was used for loading normalization, and band intensity was expressed relative to the corresponding loading-control signal. WB experiments were repeated three times.

1.10 **Biosafety Evaluation**

Body mass was measured once weekly during the 6-week experiment. To reduce short-term variation caused by food intake, mice were fasted for 2 h before weighing on a Mettler Toledo ME104E balance (Switzerland). Histological safety was additionally examined after administration of ZIF-90@PU at 10, 20, or 40 mg/kg, as shown in Figure S2G. At study termination, heart, liver, spleen, lung, and kidney tissues were collected, fixed in 4% paraformaldehyde for 24 h, processed through graded ethanol and xylene, embedded in paraffin, sectioned at 5 μm, and evaluated after H&E staining by light microscopy.

Systemic biochemical safety was assessed in serum collected immediately after euthanasia.

Blood was allowed to clot for 30 min at room temperature and centrifuged for 10 min at 3000 rpm. ALT and AST were measured as hepatic indices, BUN and CREA as renal indices, and LDH and CK as cardiac-related indices. Measurements were performed on a Hitachi 7600 automated biochemical analyzer (Japan) with commercial assay kits.

1.11 **H&E Staining**

Femoral architecture was evaluated histologically on H&E-stained sections. Bones were fixed for 24 h in 4% paraformaldehyde, processed through dehydration and paraffin embedding, and cut into 5 μm sections before staining with kit C0105S (Beyotime, China). Trabecular organization and cortical thickness were examined by light microscopy. ImageJ was used to quantify BV/TV and BS.

1.12 **Three-Point Bending Test**

Mechanical properties of the right femur were determined by three-point bending with a Discovery HR-2 rheometer (TA-HR-2, TA Instruments). Samples were tested over a 2 mm support span at 2 mm/min to a maximum displacement of 1 mm. Maximum load and maximum deflection were derived from the resulting load-deflection curves.

1.13 **TRAP Activity Staining**

For histological quantification of osteoclasts, femora were fixed in 4% paraformaldehyde for 24 h and decalcified for 4 weeks in 10% EDTA at pH 7.4 (E6758, Sigma-Aldrich, USA). Tissues were then dehydrated in graded ethanol, paraffin-embedded, and cut at 5 μm. Sections were incubated with TRAP staining solution for 1 h at room temperature. TRAP-positive multinucleated osteoclasts were counted in five randomly selected microscopic fields, and the final value represented the average of counts obtained independently by two investigators.

1.14 **Immunohistochemical (IHC) Staining**

Paraffin sections for IHC were deparaffinized, subjected to antigen retrieval, and treated with 3% hydrogen peroxide (1.08597, Sigma-Aldrich, USA) to block endogenous peroxidase. A 30-min incubation in 5% BSA was used for blocking. Sections were then incubated overnight at 4°C with collagen I (1:200; ab88147, Abcam, UK), NFATc1 (1:200; PA5-90432, Thermo Fisher, USA), or MMP9 (1:200; MA5-15886, Thermo Fisher, USA) primary antibody. HRP-conjugated secondary antibody (1:500; ab205718, Abcam, UK) was applied for 30 min at 37°C, followed by DAB visualization (ab64238, Abcam, UK) and hematoxylin counterstaining. Positive staining intensity was quantified from light-microscopy images.

1.15 **Isolation and Sample Preparation of Bone Marrow CD11b^+^ Cells**

For scRNA-seq, CD11b+ marrow cells were obtained from one OVX control mouse and one ZIF-90@PU-treated mouse. Immediately after euthanasia, femora and tibiae were harvested and flushed with sterile PBS. The marrow suspension was passed through a 70 μm strainer, treated for 5 min with RBC lysis buffer (11814389001, Sigma-Aldrich, USA), and centrifuged at 300 × g for 5 min at 4°C to obtain the cell preparation used for enrichment.

CD11b+ cells were enriched magnetically. Bone marrow cells were suspended in MACS buffer composed of PBS (pH 7.2), 0.5% BSA, and 2 mM EDTA (Miltenyi Biotec, Germany), incubated with CD11b MicroBeads (30-097-142, Miltenyi Biotec, Germany) for 15 min at 4°C in darkness, and centrifuged at 300 × g for 5 min. Cells were then loaded onto an MS magnetic column. While the column remained in the magnetic field it was washed three times; removal from the magnet allowed elution of the retained CD11b+ fraction with 5 mL MACS buffer.

Flow cytometry was used to verify enrichment purity. Recovered cells were suspended in FACS buffer, stained for 30 min at 4°C in darkness with CD11b-FITC (101206, BioLegend, USA), and acquired on a BD FACSCanto II cytometer (BD Biosciences, USA). Cytometry data were analyzed with FlowJo v10 (Tree Star, USA).

**2.Table S1. Lentiviral transfection gene silencing sequences.**

| **Name** | **shRNA Sequences (5'- 3')** |
| --- | --- |
| sh-NC (Mouse) | 5'- TTCTCCGAACGTGTCACGT-3' |
| sh-LDHA-1 (Mouse) | 5'- GTTCCCAGTTAAGTCGTATAA -3' |
| sh- LDHA-2 (Mouse) | 5'- CGTGAACATCTTCAAGTTCAT -3' |
| sh- LDHA-3 (Mouse) | 5'- GCTCCCCAGAACAAGATTACA-3' |

**3.Table S2. RT-qPCR primer sequence.**

| **Gene name** | **Primer sequence** |
| --- | --- |
| Ldha (Mouse) | Forward: 5'- ACGCAGACAAGGAGCAGTGGAA -3' |
|  | Reverse: 5'- ATGCTCTCAGCCAAGTCTGCCA -3' |
| Gapdh (Mouse) | Forward: 5'- CATCACTGCCACCCAGAAGACTG -3' |
|  | Reverse: 5'- ATGCCAGTGAGCTTCCCGTTCAG -3' |

**4.Table S3. Western blot antibody information.**

| **Target name** | **Manufacturer** | **Item number** | **Dilution ratio** |
| --- | --- | --- | --- |
| NFATc1 (Rabbit) | Thermo Fisher | PA5-90432 | 1:1000 |
| TRAP (Rabbit) | Abcam | ab2391 | 1:1000 |
| CTSK (Rabbit) | Abcam | ab187647 | 1:2000 |
| MMP9 (Rabbit) | Abcam | ab283575 | 1:1000 |
| ATP6V0D2 (Rabbit) | Abcam | ab321809 | 1:1000 |
| H3K18la (Rabbit) | Cell signaling | 13998 | 1:1000 |
| pan-Kla (Rabbit) | PTM BIO | PTM-1401 | 1:1000 |
| Histone H3 (Rabbit) | Cell signaling | 9715 | 1:1000 |
| RUNX2 (Rabbit) | Abcam | ab192256 | 1:1000 |
| JUNB (Rabbit) | Abcam | ab128878 | 1:200 |
| COL1A1 (Rabbit) | Abcam | ab270993 | 1:1000 |
| LDHA (Rabbit) | Abcam | ab52488 | 1:5000 |
| α-Tubulin (Mouse) | Abcam | ab7291 | 1:5000 |

Note: Antibodies purchased from Abcam, UK or Thermo Fisher, USA, or PTM BIO, China, or Cell signaling, USA.
