# Supplementary material for: Puerarin‑Loaded pH‑Responsive ZIF‑90 Nanoparticles Ameliorate Osteoporosis via Enhancing H3K18 Lactylation and Suppressing Aberrant Osteoclast Activation
Source: Adv Sci (Weinh). 2026 Sep 27:e77869. Online ahead of print. doi: 10.1002/advs.77869 (PMC13616396; doi:10.1002/advs.77869)
Supplement: Supplementary file 2 [file ADVS-9999-e77869-s001.zip › advs77869-sup-0002-Figures/advs77869-sup-0001-S1.pdf]

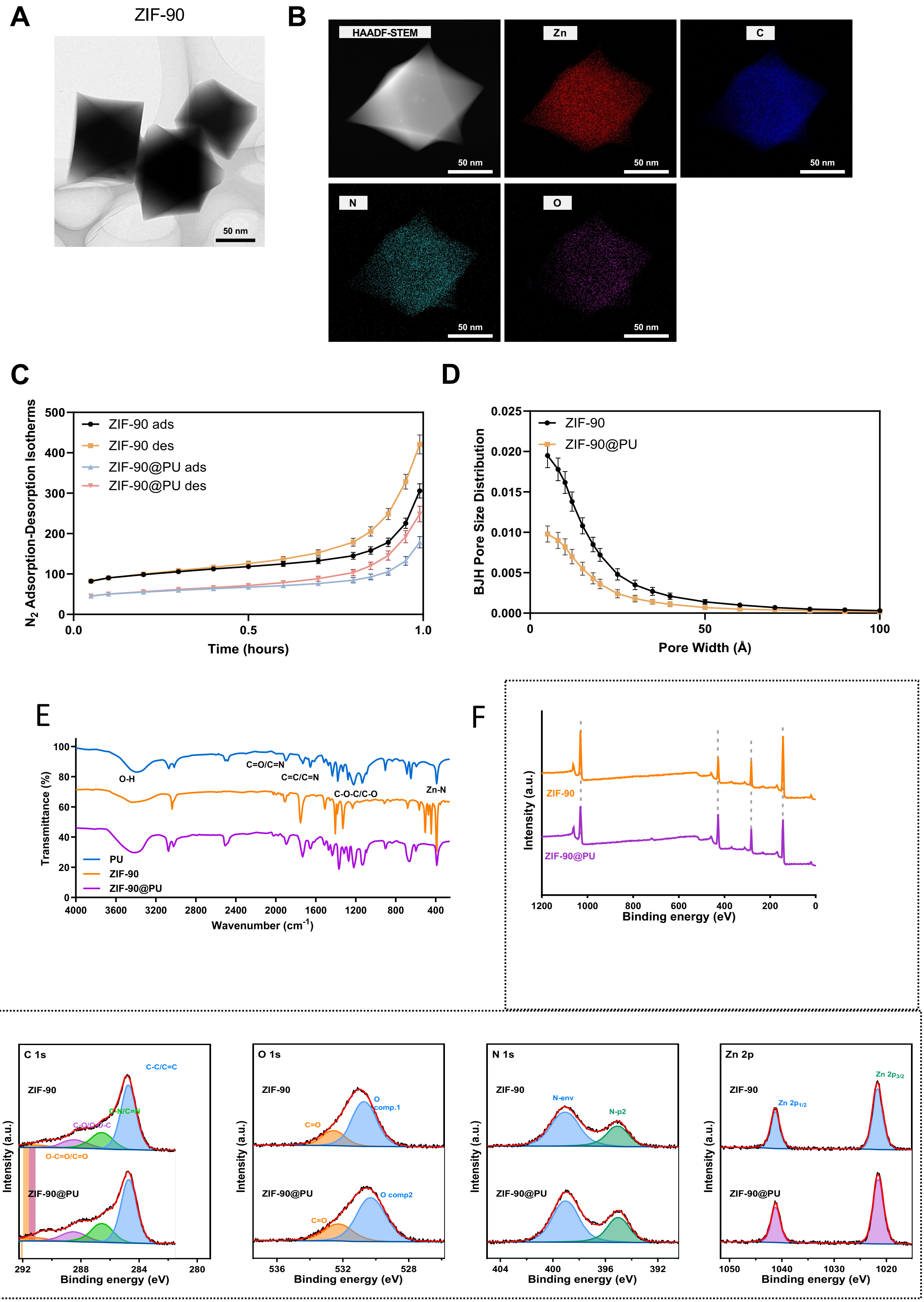
A
ZIF-90
50 nm
B
HAADF-STEM
Zn
C
N
O
50 nm
C
N_2 Adsorption-Desorption Isotherms
ZIF-90 ads
ZIF-90 des
ZIF-90@PU ads
ZIF-90@PU des
Time (hours)
D
BJH Pore Size Distribution
ZIF-90
ZIF-90@PU
Pore Width (Å)
E
Transmittance (%)
O-H
C=O/C=N
C=C/C=N
C-O-C/C-O
Zn-N
PU
ZIF-90
ZIF-90@PU
Wavenumber (cm^{-1})
F
Intensity (a.u.)
ZIF-90
ZIF-90@PU
Binding energy (eV)
C 1s
C-C/C=C
C-N/C=N
C-O/C-O-C
O-C=O/C=O
O 1s
C=O
O comp.1
O comp2
N 1s
N-env
N-p2
Zn 2p
Zn $2p_{1/2}$
Zn $2p_{3/2}$
Binding energy (eV)
